# Supplementary material for: MORC2 is a phosphorylation-dependent DNA compaction machine
Source: Nat Commun. 2025 Jul 1;16:5606. doi: 10.1038/s41467-025-60751-z (PMC12216690; doi:10.1038/s41467-025-60751-z)
Supplement: Supplementary file 1 — Supplementary Information [file 41467_2025_60751_MOESM1_ESM.pdf]

Supplementary Information for

MORC2 is a phosphorylation-dependent DNA compaction machine

Winnie Tan[1,2,3], Jeongveen Park[4], Hariprasad Venugopal[5,10], Jieqiong Lou[6,10], Prabavi Shayana Dias[7,10], Pedro L. Baldoni[1,2,10], Kyoung-Wook Moon[4,10], Toby A. Dite[1,2,10], Christine R. Keenan[1,2,6], Alexandra D. Gurzau[1,2], Joonyoung Lee[4], Timothy M Johanson[1,2], Andrew Leis[1,2], Jumana Yousef[1,2], Vineet Vaibhav[1,2], Laura F. Dagley[1,2], Ching-Seng Ang[8], Laura D. Corso[1,2], Chen Davidovich[5], Stephin J. Vervoort[1,2], Gordon K. Smyth[1,9], Marnie E. Blewitt[1,2], Rhys S. Allan[1,2], Elizabeth Hinde[6], Sheena D'Arcy[7], Je-Kyung Ryu[4,*], Shabih Shakeel[1,2,3,6,*]

[1]WEHI, 1G Royal Parade, Parkville, VIC 3052 Australia

[2]Department of Medical Biology, The University of Melbourne, Melbourne, VIC 3052 Australia

[3]ARC Centre for Cryo-electron Microscopy of Membrane Proteins, Bio21 Molecular Science and Biotechnology Institute, University of Melbourne, Parkville, Victoria, Australia

[4]Department of Physics and Astronomy, Seoul National University, Republic of Korea

[5]Department of Biochemistry and Molecular Biology, Monash University, Clayton, VIC 3168 Australia

[6]Department of Biochemistry and Pharmacology, The University of Melbourne, Melbourne, VIC 3052, Australia

[7]Department of Chemistry and Biochemistry, The University of Texas at Dallas, Texas, 75080, USA

[8]The Bio21 Molecular Science and Biotechnology Institute, The University of Melbourne, Melbourne, VIC 3052, Australia

[9]School of Mathematics and Statistics, The University of Melbourne, Melbourne, VIC 3010, Australia

[10]Equal contribution

[*]Correspondence to prof.love@snu.ac.kr or shakeel.s@wehi.edu.au

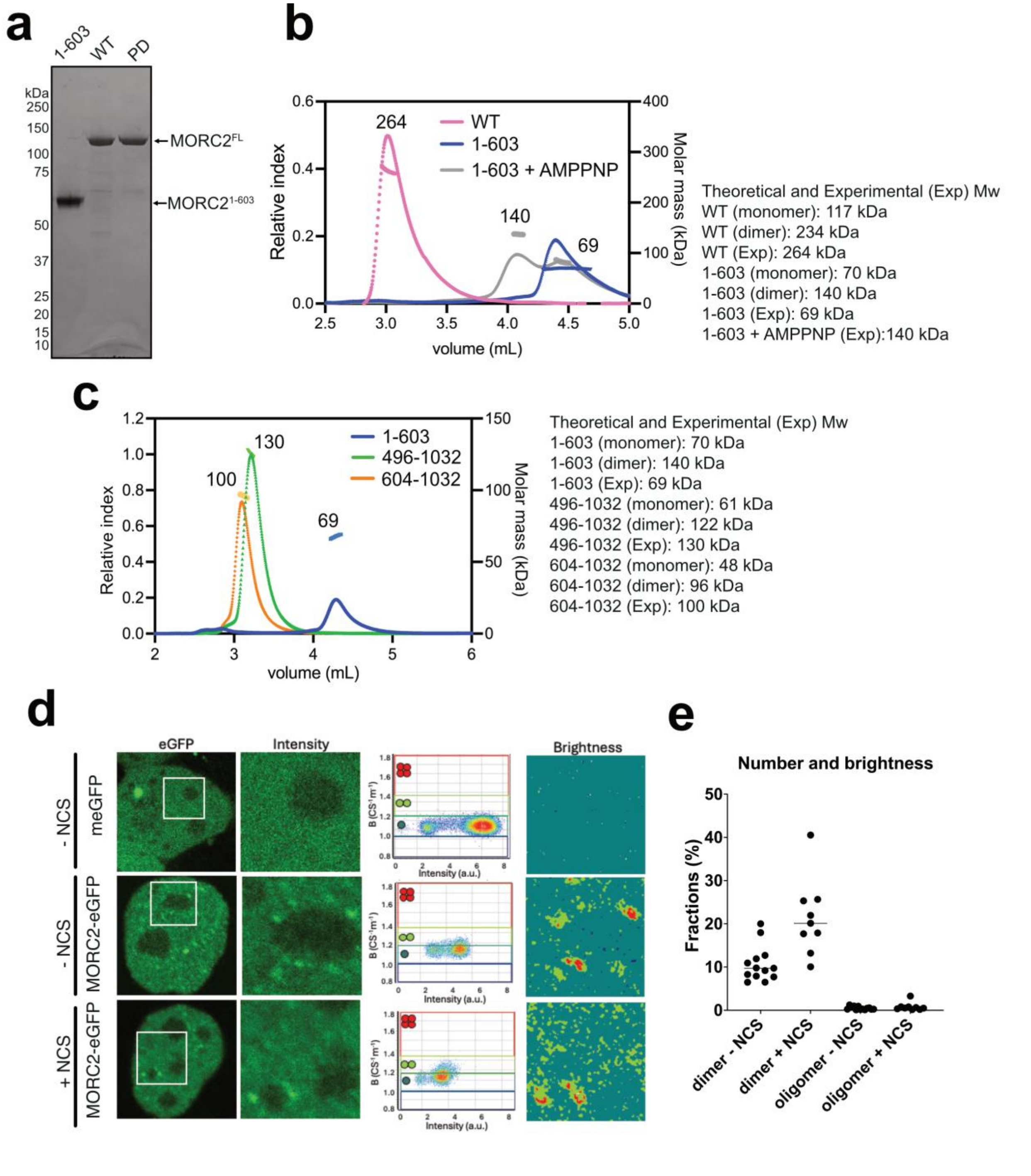

Supplementary Figure 1. MORC2 dimerises through coiled coil in its CTD.

a. Coomassie stained SDS-PAGE gel of recombinant MORC2$^{1\text{-}603}$, MORC2^{WT} and MORC2^{PD}. **b.** Size exclusion chromatography multi angle light scattering (SEC-MALS) profile of MORC2^{WT}, MORC2$^{1\text{-}603}$ with (MORC2$^{1\text{-}603+AMPPNP}$) and without AMP-PNP (MORC2$^{1\text{-}603}$) showing homodimerisation of all constructs but MORC2$^{1\text{-}603}$. **c.** SEC-MALS profile of MORC2 496-1032 construct (lacking GHKL domain) (496-1032) and 604-1032 construct (lacking GHKL and CC1) show homodimerisation while MORC2$^{1\text{-}603}$

(containing GHKL and CC1) did not. **d.** The whole nucleus containing meGFP or MORC2-eGFP is recorded. A white box is highlighted to show region of interest selected. Column 1, Intensity images of HEK293T MORC2 KO cell expressing pBacMam-meGFP (referred as “meGFP”, top), pBacMam-MORC2-meGFP (referred as “MORC2-meGFP”) in the absence (middle) or presence of neocarzinostatin (NCS, bottom). HEK293T MORC2 KO cells expressing pBacMam-meGFP were used as a monomer calibration. Column 2, The region of interest (ROI) from which a number and brightness (NB) data acquisition was recorded is shown. Column 3, The intensity versus brightness scatterplot of the NB data acquisition from column 2 with the calibrated brightness windows superimposed. Column 4, Brightness maps of the meGFP NB data acquisition presented in column 2 are coloured according to the brightness windows defined in column 3 spatially to map MORC2 monomer (teal), dimer (green) and oligomer (red) localisation. The pixel size for the number of brightness data is 41 nm. **e.** Quantification of the fraction of pixels containing MORC2 dimer and oligomer from the NB data acquisitions presented in (**d**) (Number of cells (N) $\geq$ 9 cells, two biological replicates). Mean is shown as the horizontal line. For Supp Fig 1 and e, source data are provided as a Source Data file.

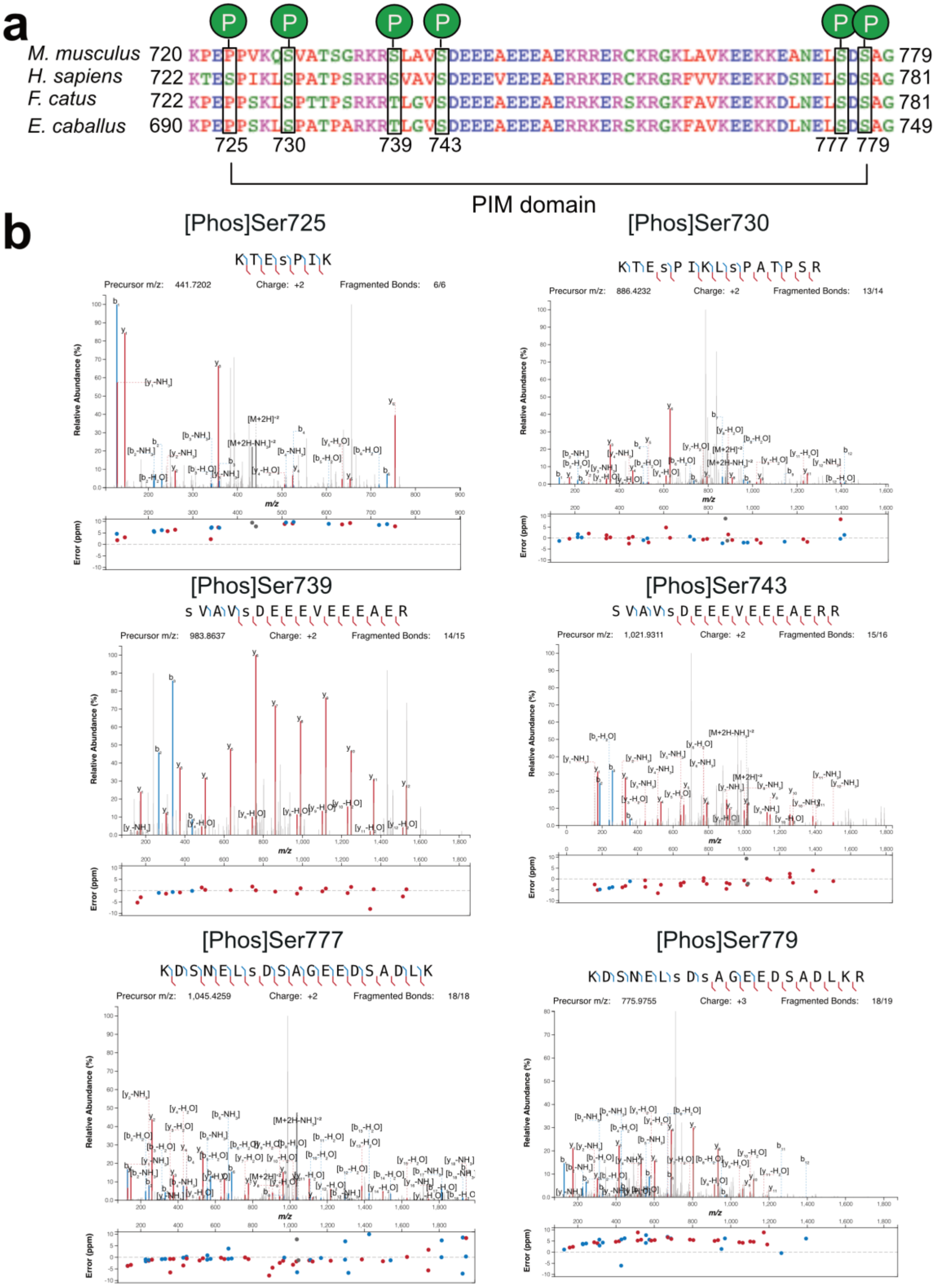
a
M. musculus 720
H. sapiens 722
F. catus 722
E. caballus 690
779
781
781
749
725
730
739
743
777
779
PIM domain
b
[Phos]Ser725
[Phos]Ser730
[Phos]Ser739
[Phos]Ser743
[Phos]Ser777
[Phos]Ser779
Relative Abundance (%)
Error (ppm)
m/z

Supplementary Figure 2. MORC2 contains multiple phosphorylation sites at the C-terminal domain.

a. Multiple sequence alignment and schematic of residues 720-781 from human MORC2 and the corresponding region in other orthologues. Highly conserved residues are boxed. The MORC2 phosphorylation sites identified in our proteomics experiments are marked with a 'P'. The phosphate interacting motif (PIM) is indicated. **b.** Annotated MS/MS spectra of phosphorylated peptides from the tryptic digest of recombinant MORC2 protein purified from insect cells.

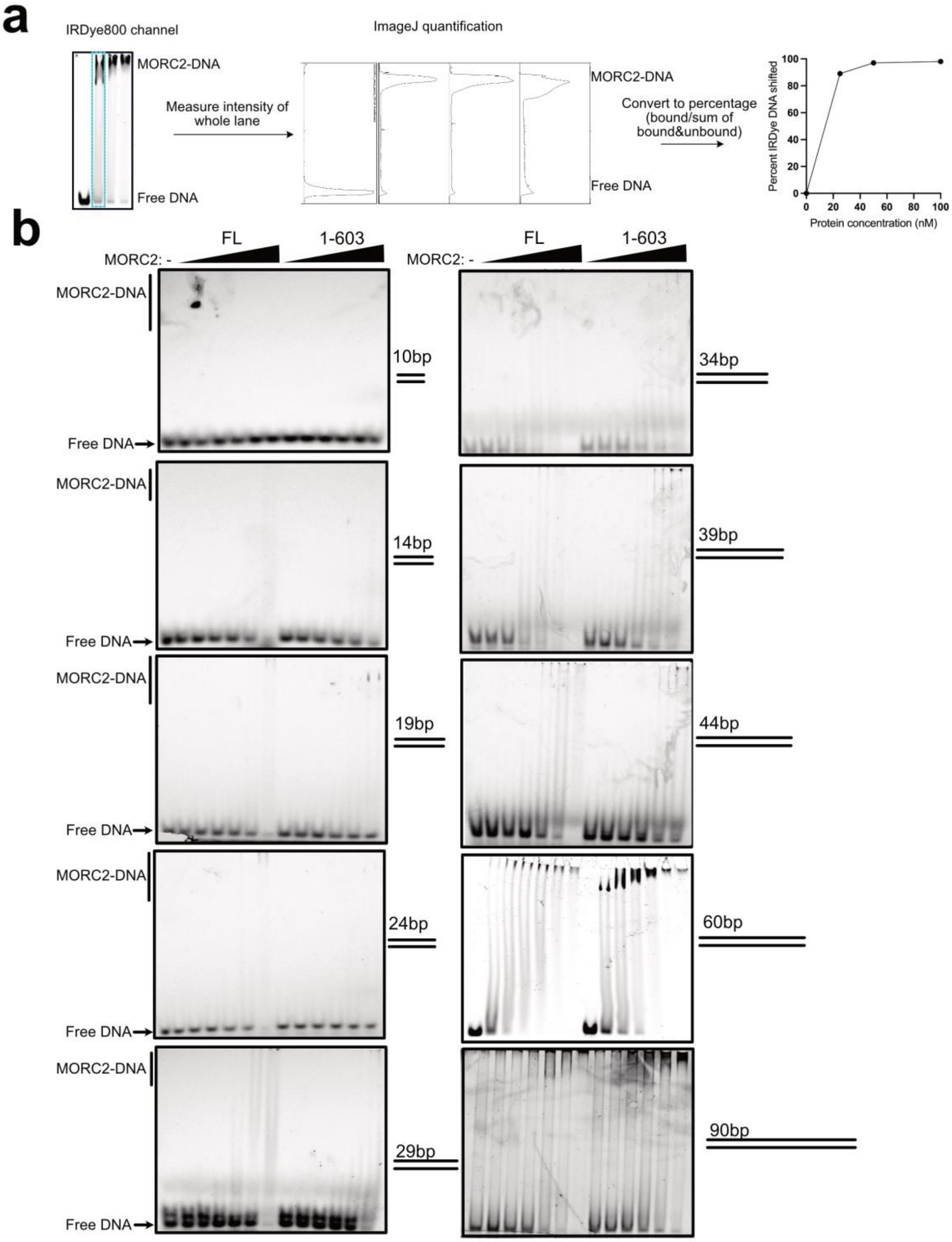
a
IRDye800 channel
MORC2-DNA
Free DNA
Measure intensity of whole lane
ImageJ quantification
MORC2-DNA
Free DNA
Convert to percentage (bound/sum of bound&unbound)
Percent IRDye DNA shifted
Protein concentration (nM)
0
20
40
60
80
100
b
FL
1-603
MORC2: -
MORC2-DNA
Free DNA
10bp
14bp
19bp
24bp
29bp
34bp
39bp
44bp
60bp
90bp

Supplementary Figure 3. Electromobility shift assay (EMSA) analysis of MORC2 minimal DNA binding region.

a. Method for calculating percentage DNA shifted of MORC2 for all EMSA except competition EMSA. A representative EMSA gel was visualised with IRDye800 channel (left). A blue dotted box is used to highlight region measured by ImageJ. ImageJ is used for quantification of percent DNA shifted vs total DNA (middle). The mean intensity of percent DNA shifted and standard deviation between the measurements are plotted on Prism (right).

b. EMSA gels of increasing concentrations (0, 25, 50, 100, 200, 400 and 800 nM) of full-length MORC2 (residues 1-1032) or its ATPase domain (residues 1-603) in the presence of 10, 14, 19, 24, 29, 34, 39, 44, 60 and 90 bp dsDNA at 25 nM concentration; number of independent experiments (n) = 2. Source data are provided as a Source Data file.

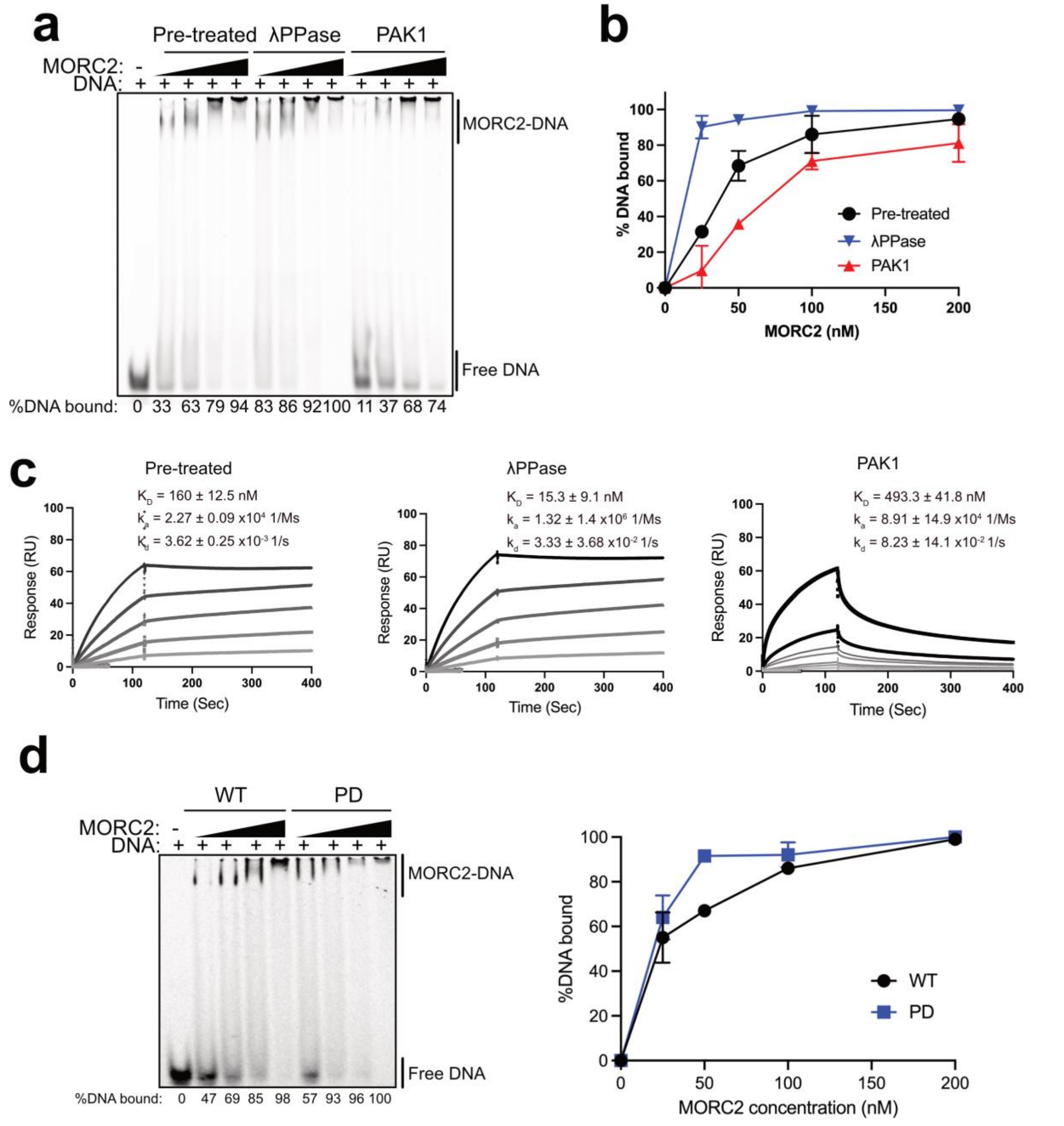

Supplementary Figure 4. Dephosphorylation of MORC2 increases its retention on DNA.

a. EMSA gels showing 0, 25, 50, 100 and 200 nM of pre-treated (WT treated with same buffer as PAK1 or λPPase), PAK1 or λPPase treated full-length MORC2 in the presence of 25 nM 60 bp IRDye-700 dsDNA. **b.** Quantification of DNA binding assays of pre-treated, λPPase and PAK1 treated MORC2. These data are shown as mean ± SD representative of experiments performed independently two times (number of independent experiments (n) = 2). **c**. Surface plasmon resonance analysis of pre-treated, λPPase and

PAK1 treated MORC2 with protein concentrations of 4, 8, 16, 33, 63, 125, 250 and 500 nM. Values for K_D (equilibrium dissociation constant), k_a (association rate constant) and k_d (dissociation rate constant) are shown as mean ± SEM, representative of three experiments. **d**. EMSA gels and quantification of percentage of 60bp IRDye-700 dsDNA (25 nM) bound to 25, 50, 100 and 200 nM of $MORC2^{WT}$ and $MORC2^{PD}$ mutant. These data are shown as mean ± SD representative of experiments performed independently two times (n=2). For Supp Fig 4, source data are provided as a Source Data file.

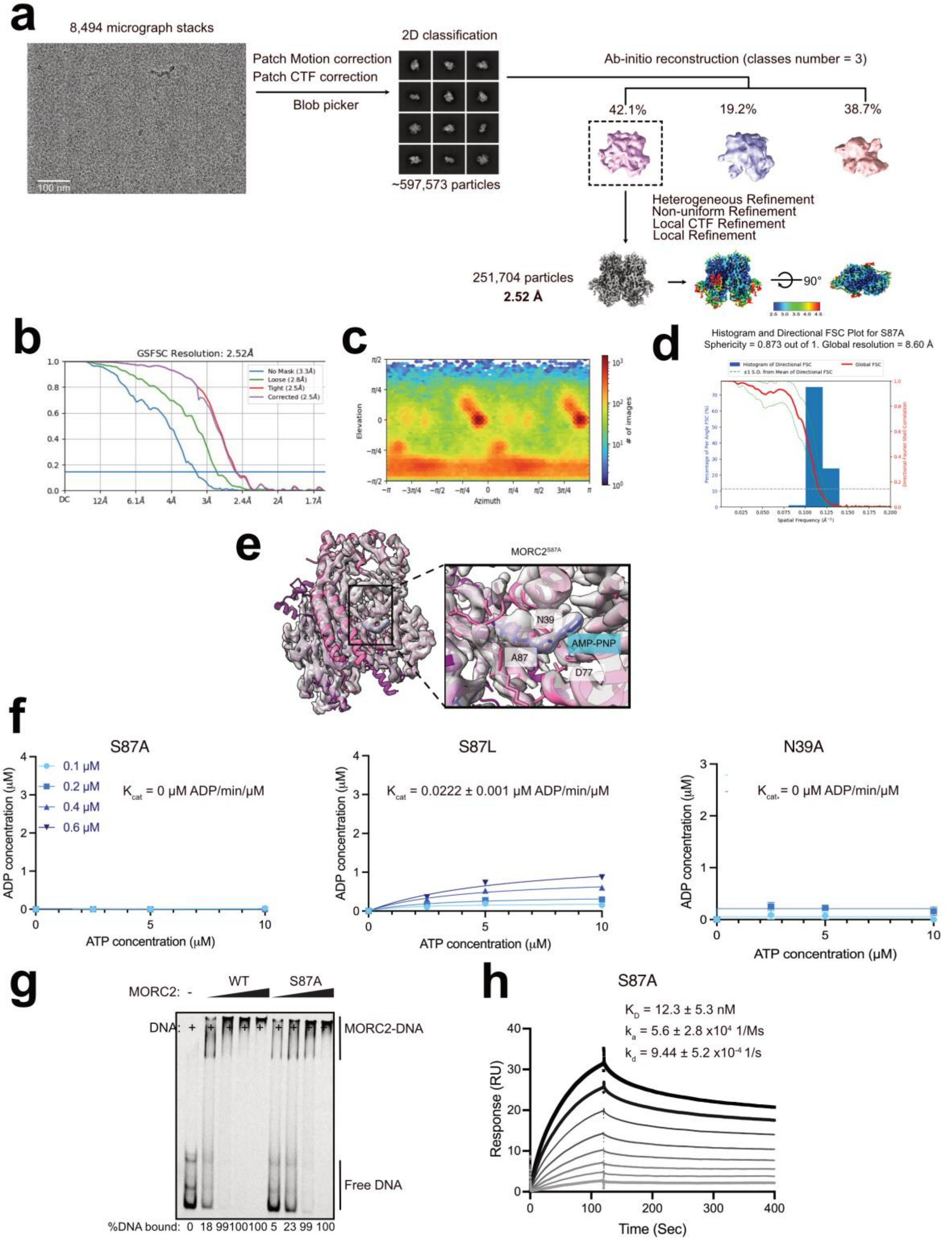

a
8,494 micrograph stacks
100 nm
Patch Motion correction
Patch CTF correction
Blob picker
2D classification
~597,573 particles
Ab-initio reconstruction (classes number = 3)
42.1%
19.2%
38.7%
Heterogeneous Refinement
Non-uniform Refinement
Local CTF Refinement
Local Refinement
251,704 particles
2.52 Å
90°
b
GSFSC Resolution: 2.52Å
No Mask (3.3Å)
Loose (2.8Å)
Tight (2.5Å)
Corrected (2.5Å)
c
Elevation
Azimuth
# of images
d
Histogram and Directional FSC Plot for S87A
Sphericity = 0.873 out of 1. Global resolution = 8.60 Å
e
MORC2S87A
N39
AMP-PNP
A87
D77
f
S87A
0.1 μM
0.2 μM
0.4 μM
0.6 μM
Kcat = 0 μM ADP/min/μM
ADP concentration (μM)
ATP concentration (μM)
S87L
Kcat = 0.0222 ± 0.001 μM ADP/min/μM
N39A
Kcat = 0 μM ADP/min/μM
g
MORC2: -
WT
S87A
DNA:
MORC2-DNA
Free DNA
%DNA bound: 0 18 99100100 5 23 99 100
h
S87A
KD = 12.3 ± 5.3 nM
ka = 5.6 ± 2.8 x10^4 1/Ms
kd = 9.44 ± 5.2 x10^-4 1/s
Response (RU)
Time (Sec)

Supplementary Figure 5. Structural and biochemical analysis of the ATPase dead, MORC2^{S87A} mutant bound to AMP-PNP.

a. Flow chart for image processing of MORC2^{S87A} as described in methods. Same processing pipeline was used for all the other cryoEM structures described in this manuscript. **b.** FSC curve of the final consensus reconstructions. **c.** Angular distribution of the particles in the final round of 3D refinement. **d.** Directional FSC plots and sphericity values calculated using the 3D-FSC server. **e.** CryoEM structure of the MORC2^{S87A} in the presence of AMP-PNP and closeup of the AMPPNP (modelled as ATP in blue) in cryoEM density. **f.** Fluorescence Polarisation ATPase assay of MORC2^{S87A}, MORC2^{S87L} and MORC2^{N39A}. Individual measurements (number of technical replicates (n) = 3) are shown, and the solid line represents the non-linear fit of the data. **g.** EMSA gel of 0, 25, 50, 100 and 200 nM of MORC2^{WT} and MORC2^{S87A} in the presence of 25 nM IRDye700-labelled dsDNA (60bp); n = 2. **h.** SPR analysis of MORC2^{S87A}. For Supp Fig 5g-h, source data are provided as a Source Data file.

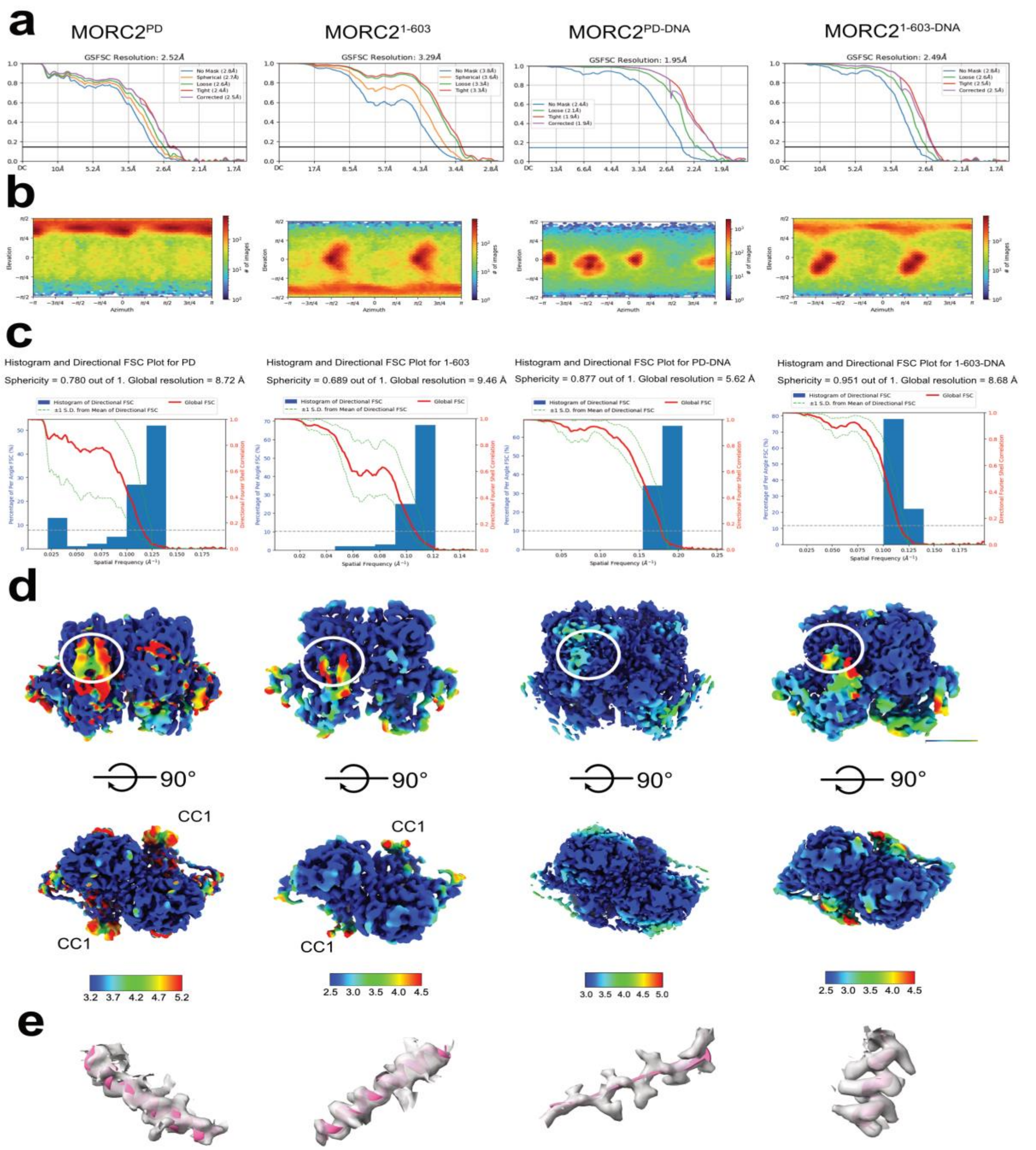

Supplementary Figure 6. CryoEM analysis of DNA bound and DNA-free MORC2^{PD} and MORC2$^{1-603}$.

a. Fourier-shell correlation (FSC) curves of the final consensus reconstructions. Reported resolutions were determined at FSC = 0.143. **b.** Angular distribution of the particles in the final round of 3D refinement. **c.**

Directional FSC plots and sphericity values calculated using the 3D-FSC server. **d.** Local resolutions (obtained in Cryosparc) plotted on the final maps. The CC1 domain of all MORC2 constructs are poorly defined in the presence of DNA (marked by white oval), suggesting that it is flexible upon DNA binding. The numbers in the colour key show local resolution range in Å. **e.** Map and model for key structural elements are shown.

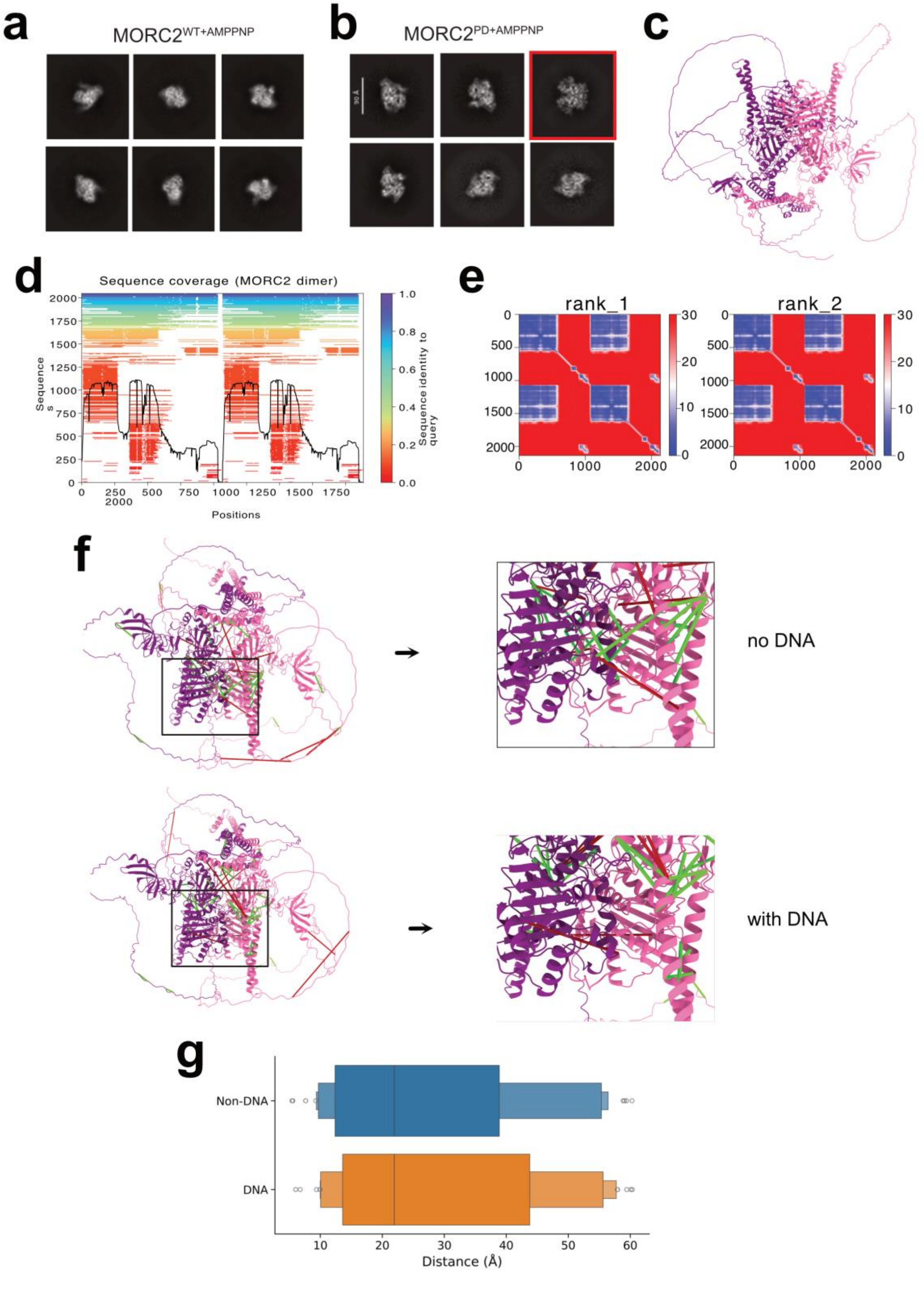
a
MORC2WT+AMPPNP
b
MORC2PD+AMPPNP
90 Å
c
d
Sequence coverage (MORC2 dimer)
Sequences
Positions
Sequence identity to query
e
rank_1
rank_2
f
no DNA
with DNA
g
Non-DNA
DNA
Distance (Å)

Supplementary Figure 7. Molecular models of MORC2.

a-b. Representative 2D classes of $MORC2^{WT}$ and $MORC2^{PD}$ in the presence of AMP-PNP. Red box highlights the MORC2 side view missing in $MORC2^{WT}$. **c-e.** AlphaFold2 structure of full-length human MORC2 dimer (residues 1-1032) Model 1with confidence metric (pLDDT) and the predicted aligned error (PAE) plots of the top 2 ranked models. **f**. Crosslinks enriched in $MORC2^{AMPPNP}$ and $MORC2^{AMPPNP\text{-}DNA}$ samples are mapped onto the full-length MORC2 (Model 1) generated by AlphaFold2. Crosslinks with distances of 20 Å are highlighted in green, while those exceeding it are marked in red. The inset highlights regions where crosslinks show the greatest differences between the $MORC2^{AMPPNP}$ and $MORC2^{AMPPNP\text{-}DNA}$ samples. **g**. A boxen plot of crosslink distances is provided for both samples ($MORC2^{AMPPNP}$ = non-DNA and $MORC2^{AMPPNP\text{-}DNA}$ = DNA). The central line is the median (50%) value. The central box contains the IQR (interquartile range) from 25 to 75%. The next box out is the 12.5%-25% percentile. The next box is 6.25–12.5%. White circles are outliers sitting outside 6.25% percentile.

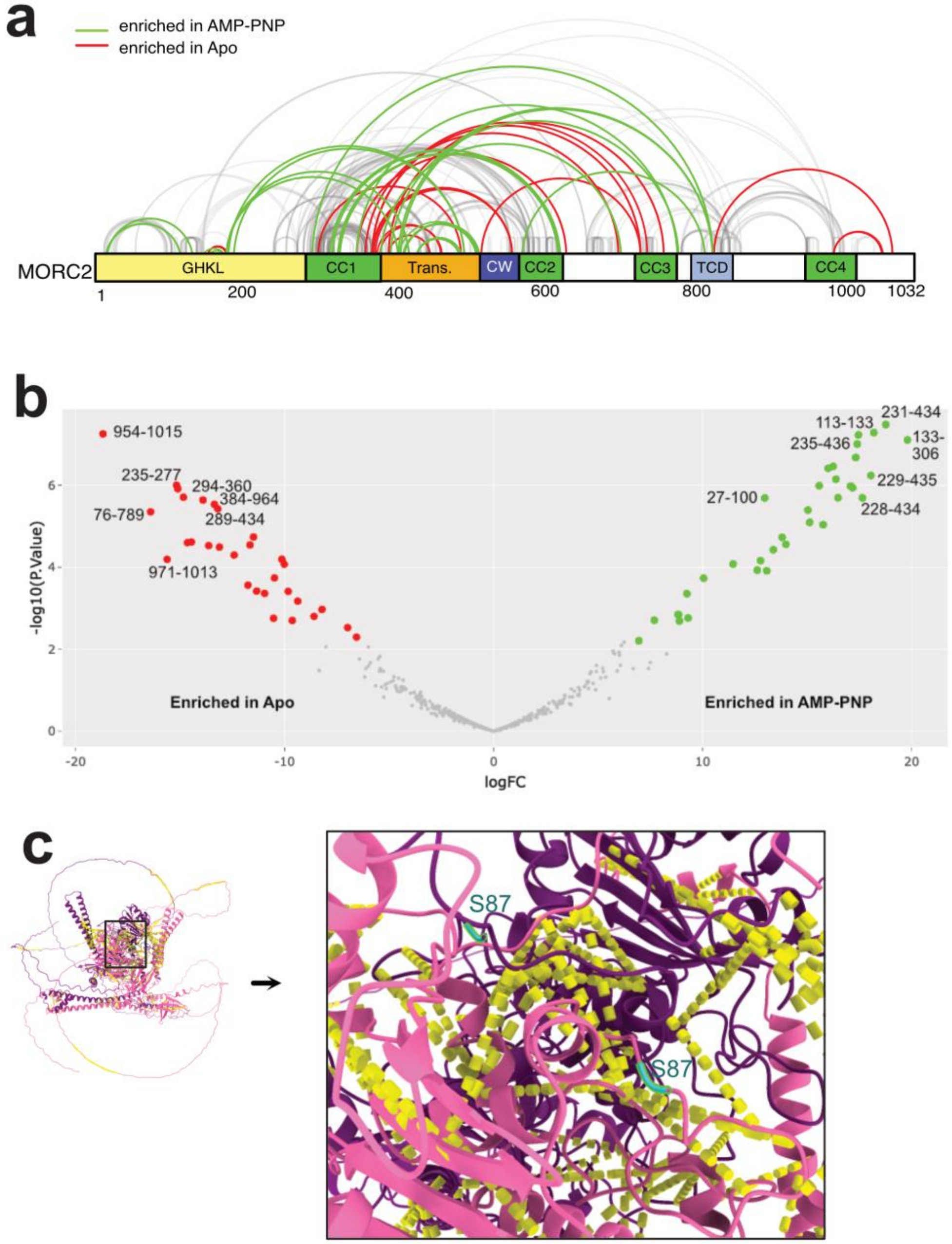
a
enriched in AMP-PNP
enriched in Apo
MORC2
GHKL
CC1
Trans.
CW
CC2
CC3
TCD
CC4
1
200
400
600
800
1000
1032
b
954-1015
235-277
294-360
384-964
76-789
289-434
971-1013
113-133
231-434
133-306
235-436
229-435
27-100
228-434
Enriched in Apo
Enriched in AMP-PNP
-log10(P.Value)
logFC
c
S87
S87

Supplementary Figure 8. Enrichment of crosslinks in MORC2 in presence and absence of AMP-PNP.

a. Map of crosslinks identified in the MORC2^{Apo} and MORC2$^{AMP\text{-}PNP}$. Green crosslinks represent crosslinks considered significantly enriched (adjusted *p*-value ≤ 0.05) in MORC2^{AMPPNP}, red crosslinks represent crosslinks enriched in MORC2^{Apo}, and grey crosslinks represent common crosslinks present in both samples. **b.** Volcano plot of quantified crosslinks, where the log2 AMPPNP/Apo fold changes are plotted against the -log10 p-value. Crosslinks that were considered significantly enriched (adjusted *p*-value ≤ 0.05) in MORC2$^{AMP\text{-}PNP}$ (green) and in MORC2^{Apo} (red) are highlighted and some of the top hit peptide residues are numbered. We adjusted for multiple comparisons with Benjamini–Hochberg (BH) correction and the statistical test was two-sided moderated t-test. **c.** Crosslinked enriched in MORC2$^{AMP\text{-}PNP}$ sample mapped onto the full-length MORC2 Model 1. ATP lid containing S87 is highlighted in cyan colour. The yellow dashed lines indicate crosslinks enriched in AMP-PNP sample near the AMP-PNP binding site. The inset shows GHKL region near the AMP-PNP binding sites.

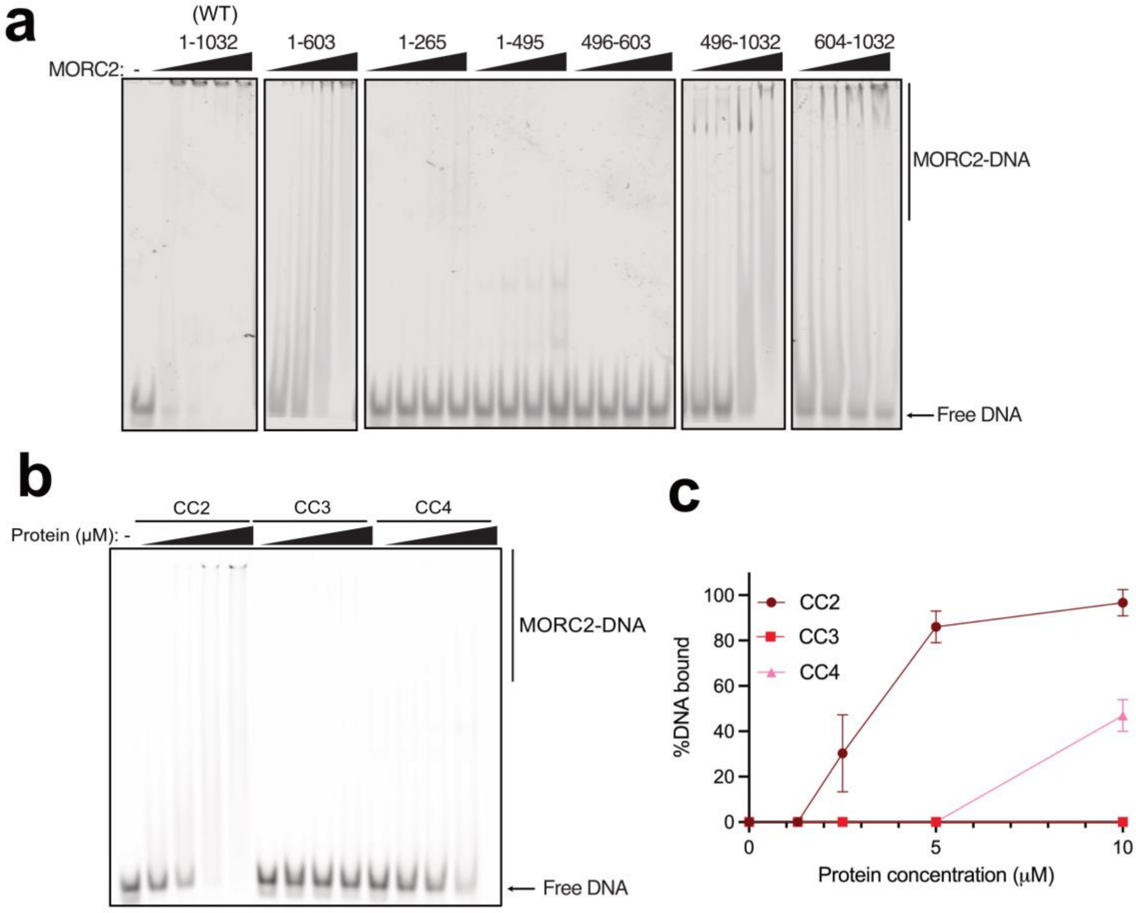

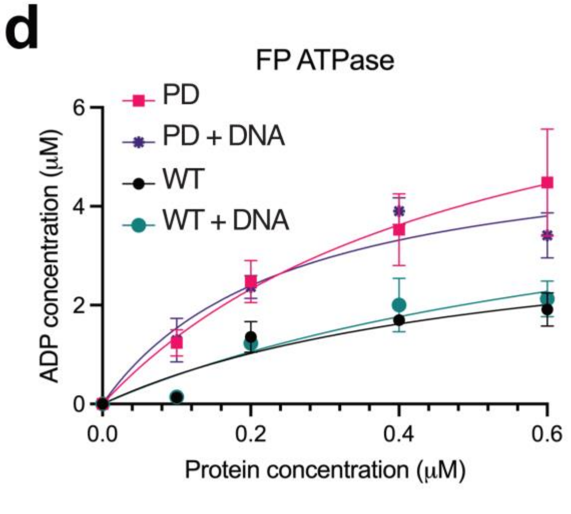

e

Reaction	K_{cat} (ADP μM/min/protein μM)
PD	0.124 ± 0.030
PD + DNA	0.093 ± 0.011
WT	0.053 ± 0.010
WT + DNA	0.059 ± 0.010

Supplementary Figure 9. DNA binding does not affect MORC2 ATPase activity despite MORC2 containing multiple DNA binding sites.

a. EMSA gels used for quantification of 25 nM of DNA binding in Fig 2d with 125, 250, 500 and 1000 nM MORC2 protein (n=3). **b.** EMSA gels showing binding of 1.3, 2.5, 5 and 10 μM of CC2, CC3 and CC4 domains to 25 nM 60 bp dsDNA. **c.** Quantification of the fraction of DNA bound for panel **b.** For each time point, individual data points are shown by their mean ± SD; number of independent experiments (n) = 3. **d.** Fluorescence Polarisation (FP) ATPase assay of 0.1, 0.2, 0.4 and 0.6 μM $MORC2^{WT}$ or $MORC2^{PD}$ mutant in the absence and presence of 0.6 μM dsDNA (60 bp). **e.** Summary of k_{cat} values is shown by their mean ± SEM representative of three experiments. For Supp Fig a-d, source data are provided as a Source Data file.

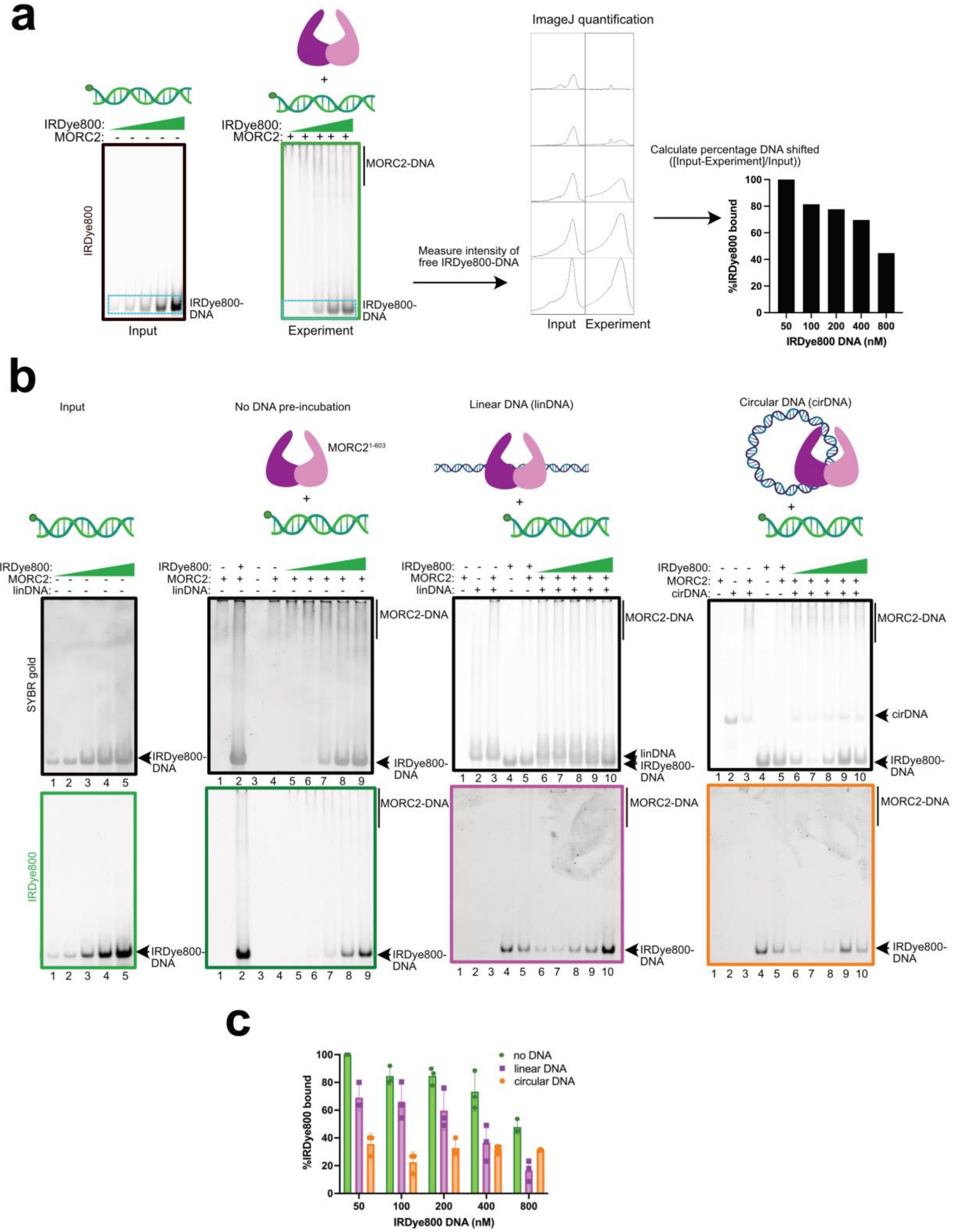
a
b
c
IRDye800:
MORC2:
IRDye800
IRDye800-DNA
Input
MORC2-DNA
Experiment
ImageJ quantification
Measure intensity of free IRDye800-DNA
Calculate percentage DNA shifted ([Input-Experiment]/Input))
%IRDye800 bound
IRDye800 DNA (nM)
No DNA pre-incubation
Linear DNA (linDNA)
Circular DNA (cirDNA)
MORC2^1-603
linDNA:
cirDNA:
SYBR gold
linDNA
cirDNA
no DNA
linear DNA
circular DNA

Supplementary Figure 10. MORC2 lacking C-terminal domain does not retain bound DNA upon competition.

a. Method for calculating percentage DNA shifted for competition EMSA. Representative EMSA gels of input (50, 100, 200, 400 and 800 nM 90bp IRDye800-labelled dsDNA only) and experimental EMSA (100 nM MORC2 incubated with 50, 100, 200, 400 and 800 nM 90bp IRDye800-labelled dsDNA for 10 min). The percentage of shifted DNA is calculated by quantifying the loss of bottom bands (experimental – input). **b.** The top panel shows schematic of competition EMSA assay with input (column 1), pre-incubated with no DNA (column 2), 101 bp linear (column 3) and 101 bp circular DNA (column 4). The 100 nM MORC2 ATPase (1-603 aa) protein was incubated with 200 nM 101 bp linear or 200 nM 101 bp circular dsDNA for 30 min at room temperature, followed by 10 min incubation with 50, 100, 200, 400 or 800 nM of 90 bp IRDye800-labelled linear dsDNA. The protein to IRDye800-labelled DNA concentration (nM) in controls (lane 2 in Column 2 and lane 5 in Column 3 and 4) is 100:800. The reaction is resolved on 6% PAGE gel. SYBR gold (top) and IRDye800 (bottom) channel images are shown. In panel a-b, The MORC2 protein and linear and circular DNA cartoons were created in BioRender. Tan, W. (2025) https://BioRender.com/kk5de6i and https://BioRender.com/q3h5kqn. **c.** Quantification of the IRDye800 shifted DNA for gels represented in (b). The points are shown as mean ± SD; number of independent experiments (n) = 3. For Supp Fig 10, source data are provided as a Source Data file.

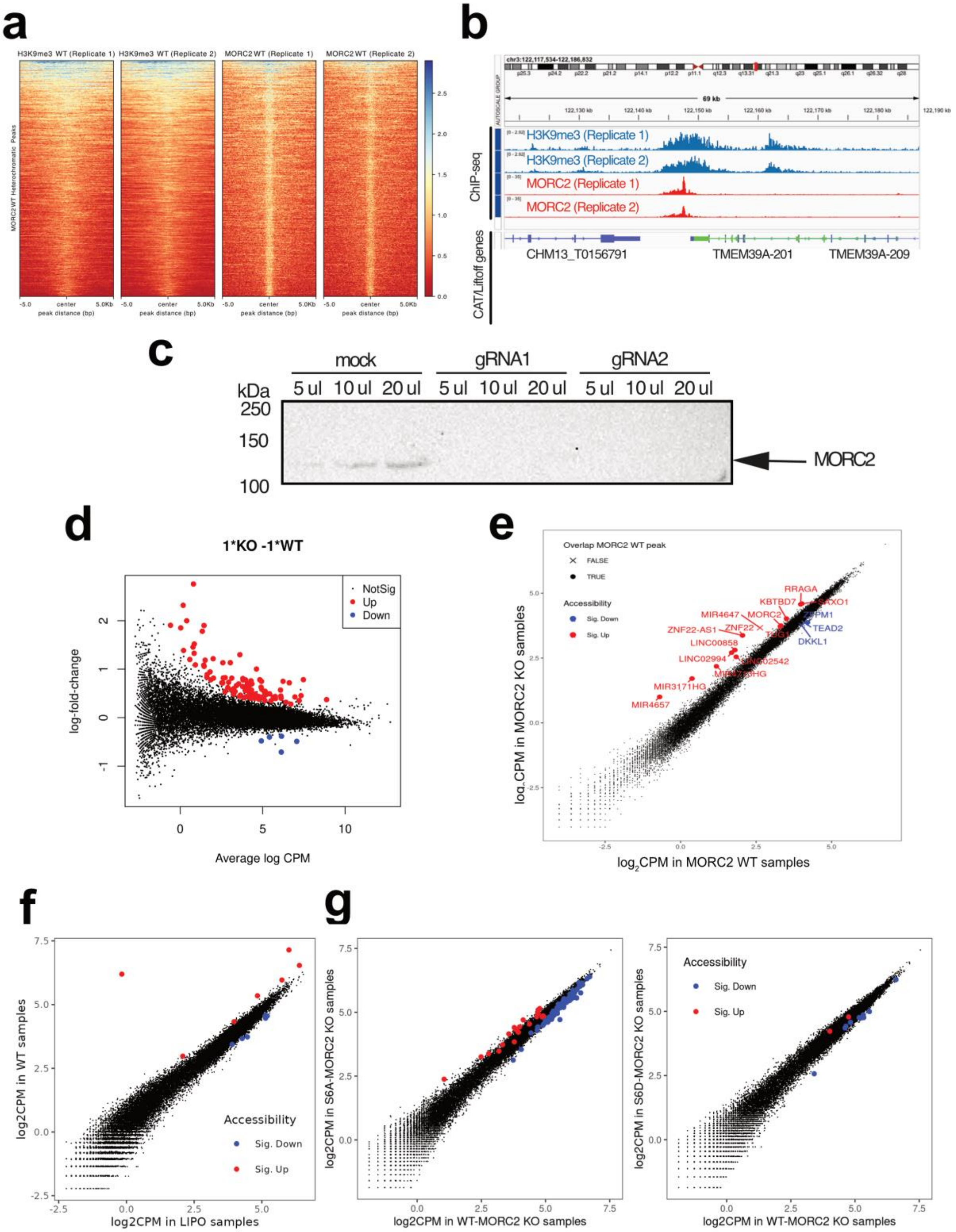

a
H3K9me3 WT (Replicate 1)
H3K9me3 WT (Replicate 2)
MORC2 WT (Replicate 1)
MORC2 WT (Replicate 2)
MORC2 WT Heterochromatic Peaks
-5.0
center
5.0Kb
peak distance (bp)
b
chr3:122,117,534-122,186,832
69 kb
H3K9me3 (Replicate 1)
H3K9me3 (Replicate 2)
MORC2 (Replicate 1)
MORC2 (Replicate 2)
ChIP-seq
CAT/Liftoff genes
CHM13_T0156791
TMEM39A-201
TMEM39A-209
c
mock
gRNA1
gRNA2
kDa
5 ul
10 ul
20 ul
250
150
100
MORC2
d
1*KO -1*WT
NotSig
Up
Down
log-fold-change
Average log CPM
e
Overlap MORC2 WT peak
FALSE
TRUE
Accessibility
Sig. Down
Sig. Up
RRAGA
KBTBD7
MIR4647
MORC2
ZNF22-AS1
ZNF22
TEAD2
DKKL1
LINC00858
LINC02994
LINC02542
MIR3171HG
MIR4657
log2CPM in MORC2 KO samples
log2CPM in MORC2 WT samples
f
log2CPM in WT samples
log2CPM in LIPO samples
Accessibility
Sig. Down
Sig. Up
g
log2CPM in S6A-MORC2 KO samples
log2CPM in WT-MORC2 KO samples
log2CPM in S6D-MORC2 KO samples
log2CPM in WT-MORC2 KO samples
Accessibility
Sig. Down
Sig. Up

Supplementary Figure 11. Genomics analysis of MORC2 and its variants show different phenotypes.

a. Heat map of ChIP-seq signal of MORC2 and H3K9me3 at MORC2 peaks outside of promoter regions (number of peaks identified = 3095). **b.** Representative IGV image of MORC2 and H3K9me3 ChIP-seq tracks where MORC2 peak coincide with H3K9me3 peak outside a promoter region (chr3:122,117,534-122,186,832). For **a-b**, two biological replicates from ChIP MORC2 and H3K9me3 assays were used for visualisation purposes. **c.** Western Blot validation of $MORC2^{KO}$ cell lines (n=2). Source data are provided as a Source Data file. **d.** Mean-Difference (MD) plot of RNA-seq analysis highlighting differentially expressed genes. **e.** Scatterplot of ATAC-seq log2 Count per million (CPM) for MORC2 WT (x-axis) and KO (y-axis) summarized at the promoter-level and averaged over biological replicates (number of biological replicates (n) = 2). Genes highlighted in red/blue are statistically up- or down-regulated in KO samples (FDR 0.05). For differentially accessible (DA) genes only, circles or crosses indicate MORC2 binding or non-binding in WT samples. **f**. Scatterplot of promoter-level ATAC-seq log2CPM for lipofectamine control (LIPO, x-axis) against MORC2-meGFP (y-axis). Genes highlighted in red/blue are statistically up- / down-regulated in HEK293T samples (FDR 0.05, n = 2). **g**. Scatterplot of promoter-level ATAC-seq log2CPM for MORC2-WT-meGFP transfected in HEK293T MORC2 KO cells (x-axis) against MORC2-S6A-meGFP (phosphodead, left) and MORC2-S6D-meGFP (phosphomimic, right) (y-axis). Genes highlighted in red/blue are statistically up- / down-regulated in HEK293T samples (FDR 0.05, n = 2).

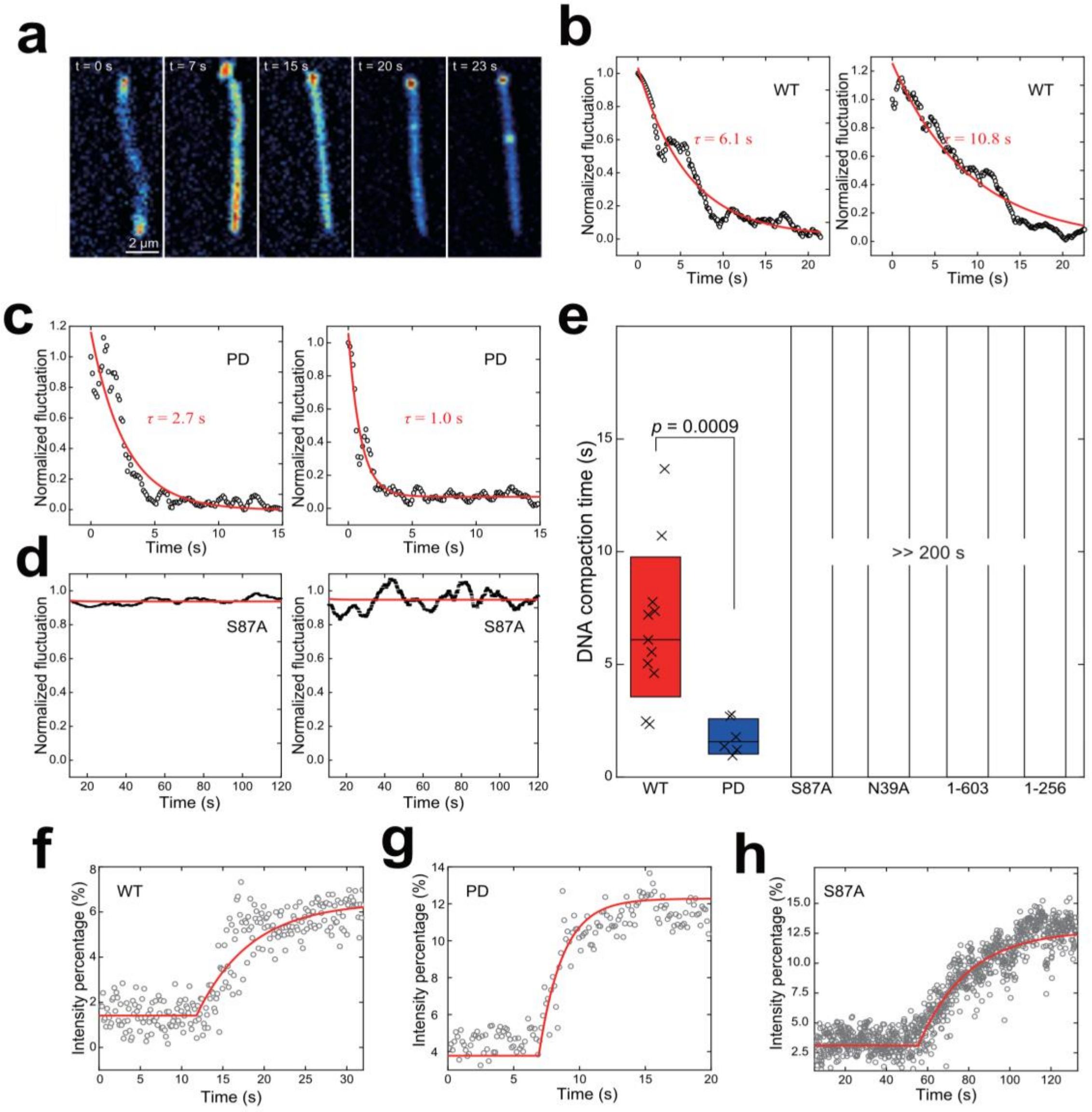

Supplementary Figure 12. MORC2-mediated DNA-compaction kinetics analysis using a double-tethered DNA.

a. Snapshots of a doubled tethered DNA losing flexibility due to MORC2-induced DNA compaction. **b–d.** Representative traces of $MORC2^{WT}$, $MORC2^{PD}$ and $MORC2^{S87A}$ fluctuation analysis in the presence of ATP. **e.** Compaction time of $MORC2^{WT}$, $MORC2^{PD}$, $MORC2^{S87A}$, $MORC2^{1–603}$ and $MORC2^{604–1032}$ and (mean ± SD, number of technical replicates (*n)* = 11, 6, 20, 20 and 20 DNA molecules for $MORC2^{WT}$, $MORC2^{PD}$, $MORC2^{S87A}$, $MORC2^{1–603}$ and $MORC2^{604–1032}$, respectively. One independent experiment for all except WT). Source data are provided as a Source Data file. **f–g**. Representative traces of $MORC2^{WT}$, $MORC2^{PD}$ and $MORC2^{S87A}$ cluster analysis. For panels **e,** the centre line and bounds of the box represent the mean and SD, respectively. The *p*-values for these panels were obtained by the two-tailed unpaired *t*-test, with no adjustments for multiple comparisons.

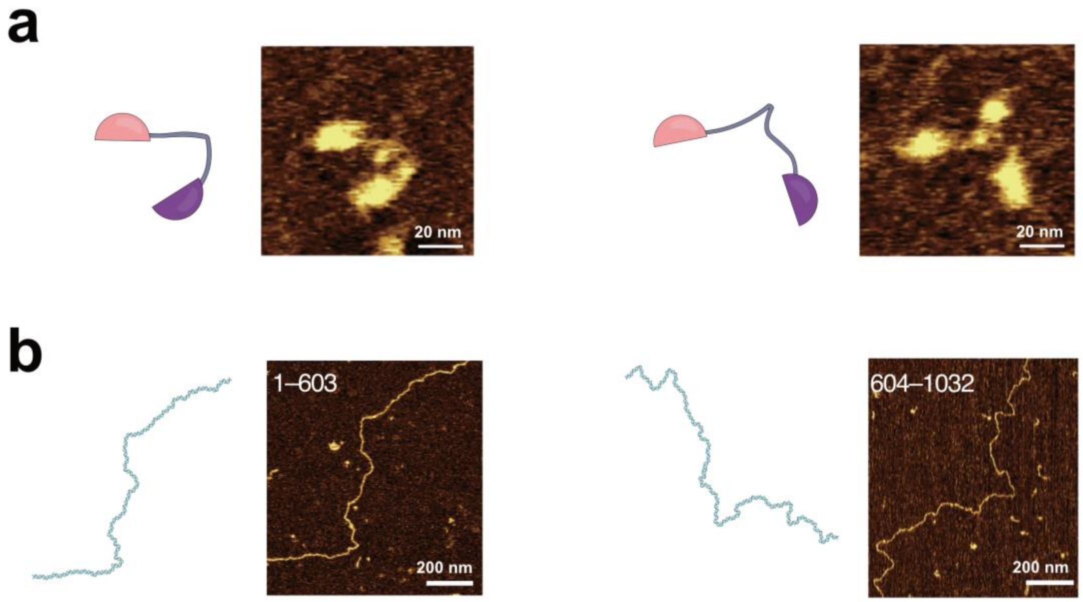

Supplementary Figure 13. AFM images of MORC2 N39A mutant, 1-603 or 604-1032 amino acids.
a. Cartoons and representative images illustrating V-shape of the MORC2^{N39A} dimer. **b.** Cartoons and representative images illustrating MORC2$^{1-603}$ and MORC2$^{604-1032}$ constructs where no DNA clamping is observed.

Supplementary Table 1. Mass spectrometry analysis of MORC2 phosphorylation from proteins purified from insect cells with ATP, PAK1 or λ-protein phosphatase treatment. Phosphopeptides highlighted in italics indicate the sites chosen for making the phosphodead mutant. UniProt id used is Q9Y6X9 and gene name used is MORC2.

**Position within MORC2**	**Phospho site**	**Phospho (STY) probabilities**	**Intensity MORC2_ATP**	**Score MORC2_ATP**	**Intensity MORC2_PAK1_ATP**	**Score MORC2_PAK1_ATP**	**Intensity MORC2_LPP**	**Score MORC2_LPP**
743	*S*	*S(1)VAVS(1)DEEEVEEEAERR*	*2.08E+09*	*329.4*	*3.16E+09*	*282.3*	*2.67E+07*	*353.9*
725	*S*	*KTES(1)PIK*	*4.16E+08*	*270*	*1.47E+09*	*284.8*	*0*	*175.3*
777	*S*	*DSNELS(1)DSAGEEDSADLKR*	*2.97E+08*	*478.2*	*1.55E+09*	*475.1*	*0*	*331.0*
779	*S*	*KDSNELS(1)DS(1)AGEEDSADLKR*	*2.97E+08*	*478.2*	*1.55E+09*	*475.1*	*0*	*331.0*
739	*S*	*S(1)VAVS(1)DEEEVEEEAERR*	*8.49E+07*	*283.8*	*1.57E+08*	*249.3*	*0*	*255.7*
730	*S*	*KTESPIKLS(1)PATPSR*	*7.53E+07*	*268.9*	*3.56E+08*	*293.1*	*0*	*175.33*
705	S	PAPLVQQLSPSLLPNS(0.021)KS(0.979)PR	1.34E+07	154.8	1.93E+07	212.8	0	0
1016	T	VQEDIDINT(1)DDELDAYIEDLITK	9.83E+06	103.7	3.16E+06	80.06	0	0
773	S	KDS(0.667)NELS(0.667)DS(0.667)AGEEDSADLKR	9.56E+06	151.4	0.00E+00	58.16	0	81.4
835	T	FDYVPT(0.037)DT(0.788)T(0.175)PR	3.66E+06	97.46	1.40E+06	103.2	0	73.4

Supplementary Table 2. CryoEM data collection, refinement and validation statistics

**Data Collection**	**MORC2^{PD} (EMD-45474)**	**MORC2$^{1\text{-}603}$ (EMD- 45477)**	**MORC2$^{PD\text{-}DNA}$ (EMD- 45476)**	**MORC2$^{1\text{-}603\text{-}DNA}$ (EMD- 45478)**	**MORC2^{S87A} (EMD- 45475)**
Micrographs	5,816	1,514	5,495	6,486	8,494
Particles (Final map)	201,271	224,700	364,243	185,490	251,704
Pixel size (Å)	0.82	1.33	0.82	0.82	0.82
Defocus range (μm)	0.5-2.0	0.5-2.0	0.5-2.0	0.5-2.0	0.5-2.0
Voltage (kV)	300	200	300	300	300
Electron dose (e/Å^2)	50	50	50	50	50
Resolution (0.143 FSC) (Å)	2.39	3.25	1.95	2.49	2.43
Final Refinement	Non-Uniform (NU) in cryosparc	NU	NU	NU	NU
Model used	5OF9	5OF9	5OF9	5OF9	5OFB
Unmodelled regions (in comparison to crystal structure)	308-342	308-342	291-364	289-364	1-3, 308-338
$CC_{map\ model}$	0.56	0.78	0.86	0.87	0.86
Model quality					
RMSD					
Bond length (Å) / Bond angles (°)	0.006/1.061	0.006/1.085	0.003/0.529	0.003/0.578	0.006/0.997
Ramachandran					
Favoured (%)	90.67	87.56	97.17	96.39	97.57

Outliers (%)	0.60	0.90	0	0.11	0
Rotamer outliers (%)	4.30	2.72	0.12	0.98	2.52
C-Beta deviations (%)	0	0	0	0	0
Clashscore	12.06	6.88	6.64	8.27	8.12
MolProbity Score	2.61	2.32	1.52	1.69	1.84

Supplementary Table 3. Table of oligonucleotides used to generate dsDNA for EMSA study.

Oligo No.	Sequence
XOm1 (60bp)	ACGCTGCCGAATTCTACCAGTGCCTTGCTAGGACATCTTTGCCCACCTGCAG GTTCACCC
XOm1.comp (60 bp)	GGGTGAACCTGCAGGTGGGCAAAGATGTCCTAGCAAGGCACTGGTAGAATT CGGCAGCGT
44-P1	ATCGATGTCTCTAGACAGCTGCTCAGGATTGATCTGTAATGGCC
39-P1	ATCGATGTCTCTAGACAGCTGCTCAGGATTGATCTGTAA
34-P1	ATCGATGTCTCTAGACAGCTGCTCAGGATTGATC
29-P1	ATCGATGTCTCTAGACAGCTGCTCAGGAT
24-P1	ATCGATGTCTCTAGACAGCTGCTC
19-P1	ATCGATGTCTCTAGACAGC
14-P1	ATCGATGTCTCTAG
10-P1	ATCGATGTCT
44-P7	GGCCATTACAGATCAATCCTGAGCAGCTGTCTAGAGACATCGAT
39-P7	TTACAGATCAATCCTGAGCAGCTGTCTAGAGACATCGAT
34-P7	GATCAATCCTGAGCAGCTGTCTAGAGACATCGAT
29-P7	ATCCTGAGCAGCTGTCTAGAGACATCGAT
24-P7	GAGCAGCTGTCTAGAGACATCGAT
19-P7	GCTGTCTAGAGACATCGAT

14-P7	CTAGAGACATCGAT
10-P7	AGACATCGAT
101bp circular dsDNA	CGGTCGTTTAGCATACTAATCTGAGAGTCCGACGGTCGTCAGTCAGTC ATGACTTCACAGAGGAAGGGCCAGTATCCTGTCCAAACTTGATGCTCG AATTC
101bp linear dsDNA	CGGTCGTTTAGCATACTAATCTGAGAGTCCGACGGTCGTCAGTCAGTC ATGACTTCACAGAGGAAGGGCCAGTATCCTGTCCAAACTTGATGCTCG AATTC
90 bp dsDNA	CGG TCG TTT AGC ATA CTA ATC TGA GAG TCC GAC GGT CGT CAG TCA GTC ATG ACT TCA CAG AGG AAG GGC CAG TAT CCT GTC CAA ACT TGA

Supplementary Table 4. HDX-MS Summary.

Experimental Conditions	MORC2$^{1-603}$, MORC2$^{1-603}$ + DNA, MORC2$^{496-1032}$, MORC2$^{496-1032}$ + DNA
HDX reaction details	50 mM Potassium Phosphate, 150 mM NaCl, pH_{read} = 7.4
HDX time course (s)	10^{-2}, 10^{-1}, 10^{0}, 10^{1} min at 20°C
HDX control samples	unlabeled MORC2$^{1-603}$ or MORC2$^{496-1032}$
Replicates (technical)	3 or 4
MORC2$^{1-603}$	
# of peptides	155
Sequence coverage	84.60%
Average peptide length / Redundancy	11.95 / 3.40
Calculated ΔHDX significance threshold*	0.31 Da / 2.69%
Mean Back-exchange (% of possible deuterium uptake)	37.50%
MORC2$^{496-1032}$	
# of peptides	116
Sequence coverage	80.10%
Average peptide length / Redundancy	11.53 / 2.90
Calculated ΔHDX significance threshold*	0.29 Da / 2.71%
Mean Back-exchange (% of possible deuterium uptake)	36.40%
