# Supplementary material for: MORC2 is a phosphorylation-dependent DNA compaction machine
Source: Nat Commun. 2025 Jul 1;16:5606. doi: 10.1038/s41467-025-60751-z (PMC12216690; doi:10.1038/s41467-025-60751-z)
Supplement: Supplementary file 2 — Description of Additional Supplementary Files [file 41467_2025_60751_MOESM2_ESM.pdf]

# Description of Additional Supplementary Files

**File Name: Supplementary Data 1**

**Description:** List of crosslinks before Skyline quantification, unique crosslinks for MORC2Apo, MORC2AMPPNPvsApo, MORC2AMPPNPvsDNA and MORC2AMPPNP-DNA, unique and all crosslinks for MORC2AMPPNP and MORC2AMPPNP-DNA used in XMAS.

**File Name: Supplementary Data 2**

**Description:** Summary table and raw HDX data.

**File Name: Supplementary Movie 1**

**Description:** Movie of MORC2 crosslinks enriched in non-DNA sample mapped onto AlphaFold2 structure.

**File Name: Supplementary Movie 2.**

**Description:** Movie of MORC2 crosslinks enriched in DNA sample mapped onto AlphaFold2 structure.

**File Name: Supplementary Movie 3.**

**Description:** Real-time movie of DNA compaction by MORC2WT.

**File Name: Supplementary Movie 4.**

**Description:** Real-time movie of DNA compaction by MORC2PD.

**File Name: Supplementary Movie 5.**

**Description:** Real-time movie of side-flow DNA compaction by MORC2WT.

**File Name: Supplementary Movie 6.**

**Description:** Real-time movie of DNA compaction by MORC2S87A.

**File Name: Supplementary Movie 7.**

**Description:** Real-time movie of DNA compaction by MORC2N39A.

**File Name: Supplementary Movie 8.**

**Description:** Real-time movie of DNA compaction by MORC21–603.

**File Name: Supplementary Movie 9.**

**Description:** Real-time movie of DNA compaction by MORC2604–1,032.
