# Supplementary material for: MORC2 is a phosphorylation-dependent DNA compaction machine
Source: Nat Commun. 2025 Jul 1;16:5606. doi: 10.1038/s41467-025-60751-z (PMC12216690; doi:10.1038/s41467-025-60751-z)
Supplement: Supplementary file 14 — Reporting Summary [file 41467_2025_60751_MOESM14_ESM.pdf]

nature portfolio

Corresponding author(s): Je-Kyung Ryu and Shabih Shakeel

Last updated by author(s): May 6, 2025

# Reporting Summary

Nature Portfolio wishes to improve the reproducibility of the work that we publish. This form provides structure for consistency and transparency in reporting. For further information on Nature Portfolio policies, see our Editorial Policies and the Editorial Policy Checklist.

## Statistics

For all statistical analyses, confirm that the following items are present in the figure legend, table legend, main text, or Methods section.

| n/a | Confirmed | |
|---|---|---|
| ☐ | ☒ | The exact sample size (*n*) for each experimental group/condition, given as a discrete number and unit of measurement |
| ☐ | ☒ | A statement on whether measurements were taken from distinct samples or whether the same sample was measured repeatedly |
| ☐ | ☒ | The statistical test(s) used AND whether they are one- or two-sided<br>*Only common tests should be described solely by name; describe more complex techniques in the Methods section.* |
| ☒ | ☐ | A description of all covariates tested |
| ☒ | ☐ | A description of any assumptions or corrections, such as tests of normality and adjustment for multiple comparisons |
| ☐ | ☒ | A full description of the statistical parameters including central tendency (e.g. means) or other basic estimates (e.g. regression coefficient) AND variation (e.g. standard deviation) or associated estimates of uncertainty (e.g. confidence intervals) |
| ☒ | ☐ | For null hypothesis testing, the test statistic (e.g. *F*, *t*, *r*) with confidence intervals, effect sizes, degrees of freedom and *P* value noted<br>*Give P values as exact values whenever suitable.* |
| ☒ | ☐ | For Bayesian analysis, information on the choice of priors and Markov chain Monte Carlo settings |
| ☒ | ☐ | For hierarchical and complex designs, identification of the appropriate level for tests and full reporting of outcomes |
| ☒ | ☐ | Estimates of effect sizes (e.g. Cohen's *d*, Pearson's *r*), indicating how they were calculated |

*Our web collection on statistics for biologists contains articles on many of the points above.*

## Software and code

Policy information about availability of computer code

| | |
|---|---|
| Data collection | EPU software for cryoEM data collection on Titan Krios and Arctica. BioRad Chemi Doc MP was used for gel imaging. Orbitrap Eclipse Tribrid mass spectrometer coupled with a Neo Vanquish LC and Orbitrap Eclipse™ Tribrid mass spectrometer via Easy nLC source for proteomics and crosslinking mass spectrometry. Envision plate reader (PerkinElmer Life Sciences) for Fluorescence polarization ATPase assays. Biacore S200 Instrument (Cytiva) for SPR. ChronosHDX software (Trajan) and SYNAPT G2-Si mass spectrometer (Waters) for HDX mass spectrometry. Olympus FV3000 laser scanning microscope for fluorescence microscopy. Illumina NextSeq2000 for sequencing. Olympus UPlanXApo 100x /1.45 lens and an Andor iXon Ultra 897 EM CCD for single molecule study. Multimode-8 AFM (Bruker) equipped with a Nanoscope VI controller and Nanoscope version 10.0 software for AFM. |
| Data analysis | CryoSparc, Coot, ChimeraX, Phenix, GraphPad Prism, xiSEARCH, Skyline, xiView, XMAS, MaxQuant version 2.0.1.0, Biacore S200 Evaluation Software (Cytiva), R (v4.2.1) and Bioconductor, Protein Lynx Global Server (PLGS) v3.0 (Waters), DynamX 3.0, Olympus FluoView software, SimFCS from the Laboratory for Fluorescence Dynamics (www.lfd.uci.edu), Gwyddion version 2.53. |

For manuscripts utilizing custom algorithms or software that are central to the research but not yet described in published literature, software must be made available to editors and reviewers. We strongly encourage code deposition in a community repository (e.g. GitHub). See the Nature Portfolio guidelines for submitting code & software for further information.

## Data

Policy information about availability of data

All manuscripts must include a data availability statement. This statement should provide the following information, where applicable:

- Accession codes, unique identifiers, or web links for publicly available datasets
- A description of any restrictions on data availability
- For clinical datasets or third party data, please ensure that the statement adheres to our policy

CryoEM maps have been deposited in the EM Data Bank with the following accession codes: EMD-45474 (MORC2PD) [https://www.ebi.ac.uk/pdbe/entry/emdb/EMD-45474], EMD-45477 (MORC21-603) [https://www.ebi.ac.uk/pdbe/entry/emdb/EMD-45477], EMD-45476 (MORC2PD-DNA) [https://www.ebi.ac.uk/pdbe/entry/emdb/EMD-45476], EMD-45478 (MORC21-603-DNA) [https://www.ebi.ac.uk/pdbe/entry/emdb/EMD-45478] and EMD-45475 (MORC2S87A) [https://www.ebi.ac.uk/pdbe/entry/emdb/EMD-45475]. Atomic coordinates have been deposited in the Protein Data Bank with the accession codes 9CDF (MORC2PD), 9CDI (MORC21-603), 9CDH (MORC2PD-DNA), 9CDJ (MORC21-603-DNA), and 9CDG (MORC2S87A). The raw mass spectrometry data for MORC2 phosphorylation are available from ProteomeXchange via the PRIDE partner repository with identifier PXD053383 [http://proteomecentral.proteomexchange.org/cgi/GetDataset?ID=PXD053383]; and PXD053379 [http://proteomecentral.proteomexchange.org/cgi/GetDataset?ID=PXD053379] for HDX. All crosslinking mass spectrometry data are available at JPOST with identifier JPST003183. All the ChIPseq, RNAseq and ATACseq data are deposited at GEO with accession number GSE274916 [https://www.ncbi.nlm.nih.gov/geo/query/acc.cgi?acc= GSE274916]. Source data are provided with this paper. The custom Python scripts used are deposited in Zenodo (http://doi.org/10.5281/zenodo.15347133). All other data are available in the main text or as part of the Supplementary or supplementary materials. Original gels, blot images and numerical data used to generate plots are provided in the source data. Source data are provided as a Source Data file. Correspondence and requests for materials should be addressed to JKR or SS. All unique materials are available upon request with completion of a standard Materials Transfer Agreement.

## Research involving human participants, their data, or biological material

Policy information about studies with human participants or human data. See also policy information about sex, gender (identity/presentation), and sexual orientation and race, ethnicity and racism.

| | |
|---|---|
| Reporting on sex and gender | N/A |
| Reporting on race, ethnicity, or other socially relevant groupings | N/A |
| Population characteristics | N/A |
| Recruitment | N/A |
| Ethics oversight | N/A |

Note that full information on the approval of the study protocol must also be provided in the manuscript.

# Field-specific reporting

Please select the one below that is the best fit for your research. If you are not sure, read the appropriate sections before making your selection.

☒ Life sciences ☐ Behavioural & social sciences ☐ Ecological, evolutionary & environmental sciences

For a reference copy of the document with all sections, see nature.com/documents/nr-reporting-summary-flat.pdf

# Life sciences study design

All studies must disclose on these points even when the disclosure is negative.

| | |
|---|---|
| Sample size | Sample sizes were selected based on previous experience and published studies. For DNA-binding, ATPase assays, proteomics, cross-linking and HDX mass spectrometry, ChIP-seq, ATAC-seq and RNA-seq the time points, protein and DNA concentrations and incubation times were chosen based on initial titrations and previously published data. |
| Data exclusions | No data were excluded. |
| Replication | All experiments including protein expression, purification, ATPase and DNA binding assays, crosslinking mass spectrometry, HDX mass spectrometry, FLIM-FRET and single molecule studies were performed at least three times, as indicated in the text. |
| Randomization | Randomization is not relevant to the assays and experiments performed in this study. Replicates of all experiments show the results are reproducible and are not subjected to researcher's bias. |
| Blinding | Blinding is not relevant to the experiments presented in this study. Replicates of all experiments show the results are reproducible and are not subjected to researcher's bias. All samples were processed identically under the same experimental conditions, and sample infomration did not lead to bias on any sample during analysis. |

# Reporting for specific materials, systems and methods

We require information from authors about some types of materials, experimental systems and methods used in many studies. Here, indicate whether each material, system or method listed is relevant to your study. If you are not sure if a list item applies to your research, read the appropriate section before selecting a response.

### Materials & experimental systems

| n/a | Involved in the study |
|---|---|
| ☐ | ☒ Antibodies |
| ☐ | ☒ Eukaryotic cell lines |
| ☐ | ☐ Palaeontology and archaeology |
| ☐ | ☐ Animals and other organisms |
| ☐ | ☐ Clinical data |
| ☐ | ☐ Dual use research of concern |
| ☐ | ☐ Plants |

### Methods

| n/a | Involved in the study |
|---|---|
| ☐ | ☒ ChIP-seq |
| ☐ | ☐ Flow cytometry |
| ☐ | ☐ MRI-based neuroimaging |

## Antibodies

| | |
|---|---|
| Antibodies used | anti-MORC2 Rabbit pAb (Invitrogen #PA5-51172), H3K9me3 Rabbit pAb (Abcam #8898), Rabbit IgG pAb (Abcam #ab46540) |
| Validation | We didn't conduct independent validation of the antibodies but the validation has been performed by the corresponding manufacturers and the information is available on their websites. |

## Eukaryotic cell lines

Policy information about cell lines and Sex and Gender in Research

| | |
|---|---|
| Cell line source(s) | HEK293T cells and Sf9 cells were kind gift from Glukhova lab at WEHI. |
| Authentication | We didn't authenticate these cell lines independently. |
| Mycoplasma contamination | The cell lines were not tested for mycoplasma contamination. |
| Commonly misidentified lines (See ICLAC register) | No commonly misidentified cell lines were used in this study. |

## Palaeontology and Archaeology

| | |
|---|---|
| Specimen provenance | N/A |
| Specimen deposition | N/A |
| Dating methods | N/A |

☐ Tick this box to confirm that the raw and calibrated dates are available in the paper or in Supplementary Information.

| | |
|---|---|
| Ethics oversight | *Identify the organization(s) that approved or provided guidance on the study protocol, OR state that no ethical approval or guidance was required and explain why not.* |

Note that full information on the approval of the study protocol must also be provided in the manuscript.

## Animals and other research organisms

Policy information about studies involving animals; ARRIVE guidelines recommended for reporting animal research, and Sex and Gender in Research

| | |
|---|---|
| Laboratory animals | This study did not involve animals. |
| Wild animals | N/A |
| Reporting on sex | N/A |
| Field-collected samples | N/A |

Ethics oversight | N/A

Note that full information on the approval of the study protocol must also be provided in the manuscript.

## Clinical data

Policy information about clinical studies

All manuscripts should comply with the ICMJE guidelines for publication of clinical research and a completed CONSORT checklist must be included with all submissions.

| | |
|---|---|
| Clinical trial registration | N/A |
| Study protocol | N/A |
| Data collection | N/A |
| Outcomes | N/A |

## Dual use research of concern

Policy information about dual use research of concern

### Hazards

Could the accidental, deliberate or reckless misuse of agents or technologies generated in the work, or the application of information presented in the manuscript, pose a threat to:

| No | Yes | |
|---|---|---|
| ☒ | ☐ | Public health |
| ☒ | ☐ | National security |
| ☒ | ☐ | Crops and/or livestock |
| ☒ | ☐ | Ecosystems |
| ☒ | ☐ | Any other significant area |

### Experiments of concern

Does the work involve any of these experiments of concern:

| No | Yes | |
|---|---|---|
| ☒ | ☐ | Demonstrate how to render a vaccine ineffective |
| ☒ | ☐ | Confer resistance to therapeutically useful antibiotics or antiviral agents |
| ☒ | ☐ | Enhance the virulence of a pathogen or render a nonpathogen virulent |
| ☒ | ☐ | Increase transmissibility of a pathogen |
| ☒ | ☐ | Alter the host range of a pathogen |
| ☒ | ☐ | Enable evasion of diagnostic/detection modalities |
| ☒ | ☐ | Enable the weaponization of a biological agent or toxin |
| ☒ | ☐ | Any other potentially harmful combination of experiments and agents |

## Plants

| | |
|---|---|
| Seed stocks | N/A |
| Novel plant genotypes | N/A |
| Authentication | N/A |

# ChIP-seq

## Data deposition

☒ Confirm that both raw and final processed data have been deposited in a public database such as GEO.

☒ Confirm that you have deposited or provided access to graph files (e.g. BED files) for the called peaks.

| | |
|---|---|
| Data access links<br>*May remain private before publication.* | https://www.ncbi.nlm.nih.gov/geo/query/acc.cgi?acc=GSE274916 and enter token axcxscyqftudfgz into the box |
| Files in database submission | FASTQ, bigWig and peak files provided for every sample |
| Genome browser session<br>(e.g. UCSC) | N/A |

## Methodology

| | |
|---|---|
| Replicates | All samples were run in duplicate with good agreement |
| Sequencing depth | ChIP_H3K9me3_KO_1: 88080674 reads, 58423892 uniquely mapped reads, 65 bp, paired-end<br>ChIP_H3K9me3_KO_2: 94697735 reads, 62646497 uniquely mapped reads, 65 bp, paired-end<br>ChIP_H3K9me3_WT_1: 61131813 reads, 39325019 uniquely mapped reads, 65 bp, paired-end<br>ChIP_H3K9me3_WT_2: 118640368 reads, 77797446 uniquely mapped reads, 65 bp, paired-end<br>ChIP_IgG_KO_1: 29248385 reads, 23474810 uniquely mapped reads, 65 bp, paired-end<br>ChIP_IgG_KO_2: 10046019 reads, 8232490 uniquely mapped reads, 65 bp, paired-end<br>ChIP_IgG_WT_1: 24336180 reads, 20112465 uniquely mapped reads, 65 bp, paired-end<br>ChIP_IgG_WT_2: 21876965 reads, 17651898 uniquely mapped reads, 65 bp, paired-end<br>ChIP_MORC2_KO_1: 23987031 reads, 19375363 uniquely mapped reads, 65 bp, paired-end<br>ChIP_MORC2_KO_2: 24436954 reads, 19697631 uniquely mapped reads, 65 bp, paired-end<br>ChIP_MORC2_WT_1: 31248420 reads, 25444851 uniquely mapped reads, 65 bp, paired-end<br>ChIP_MORC2_WT_2: 29671416 reads, 23988992 uniquely mapped reads, 65 bp, paired-end |
| Antibodies | anti-MORC2 Rabbit pAb (Invitrogen #PA5-51172), H3K9me3 Rabbit pAb (Abcam #8898), Rabbit IgG pAb (Abcam #ab46540) |
| Peak calling parameters | macs3 callpeak --treatment H3K9me3_KO*.bam --control IgG_KO*.bam --format BAMPE --gsize 2.7e9 --bdg --outdir output/ --name H3K9me3_KO --broad --broad-cutoff 0.1<br>macs3 callpeak --treatment H3K9me3_WT*.bam --control IgG_WT*.bam --format BAMPE --gsize 2.7e9 --bdg --outdir output/ --name H3K9me3_WT --broad --broad-cutoff 0.1<br>macs3 callpeak --treatment MORC2_KO*.bam --control IgG_KO*.bam --format BAMPE --gsize 2.7e9 --bdg --outdir output/ --name MORC2_KO<br>macs3 callpeak --treatment MORC2_WT*.bam --control IgG_WT*.bam --format BAMPE --gsize 2.7e9 --bdg --outdir output/ --name MORC2_WT |
| Data quality | Quality control was performed with FASTQC v0.12.1 to evaluate number of sequence reads, quality scores, GC content, sequence length distribution, sequence duplication, and adapter content.<br>H3K9me3_KO: 101859 peaks, 58 peaks above 5-fold enrichment<br>H3K9me3_WT: 116550 peaks, 138 peaks above 5-fold enrichment<br>MORC2_KO: 1802 peaks, 610 peaks above 5-fold enrichment<br>MORC2_WT: 15366 peaks, 4162 peaks above 5-fold enrichment |
| Software | Quality control was performed with FASTQC v0.12.1. Adaptor trimming was performed with trim galore v0.6.10. Read filtering was performed with samtools v1.18 and Picard-tools v2.26.11. Alignment was performed with bowtie2 v2.4.4. Peak calling was performed with MACS3 v3.0.0b3. Coverage tracks and plots were generated with deepTools v.3.5.1. |

# Flow Cytometry

## Plots

Confirm that:

☐ The axis labels state the marker and fluorochrome used (e.g. CD4-FITC).

☐ The axis scales are clearly visible. Include numbers along axes only for bottom left plot of group (a 'group' is an analysis of identical markers).

☐ All plots are contour plots with outliers or pseudocolor plots.

☐ A numerical value for number of cells or percentage (with statistics) is provided.

## Methodology

| | |
|---|---|
| Sample preparation | N/A |

Instrument | N/A
Software | N/A
Cell population abundance | N/A
Gating strategy | N/A

☐ Tick this box to confirm that a figure exemplifying the gating strategy is provided in the Supplementary Information.

## Magnetic resonance imaging

### Experimental design

Design type | N/A
Design specifications | N/A
Behavioral performance measures | N/A

### Acquisition

Imaging type(s) | N/A
Field strength | N/A
Sequence & imaging parameters | N/A
Area of acquisition | N/A

Diffusion MRI ☐ Used ☒ Not used

### Preprocessing

Preprocessing software | N/A
Normalization | N/A
Normalization template | N/A
Noise and artifact removal | N/A
Volume censoring | N/A

### Statistical modeling & inference

Model type and settings | N/A
Effect(s) tested | N/A

Specify type of analysis: ☐ Whole brain ☐ ROI-based ☐ Both

Statistic type for inference (See Eklund et al. 2016) | N/A

Correction | N/A

### Models & analysis

| n/a | Involved in the study |
|---|---|
| ☒ | ☐ Functional and/or effective connectivity |
| ☒ | ☐ Graph analysis |
| ☒ | ☐ Multivariate modeling or predictive analysis |
