# Supplementary material for: MORC2 is a phosphorylation-dependent DNA compaction machine
Source: Nat Commun. 2025 Jul 1;16:5606. doi: 10.1038/s41467-025-60751-z (PMC12216690; doi:10.1038/s41467-025-60751-z)

Unmodified SDS-PAGE of Supp Fig 1a

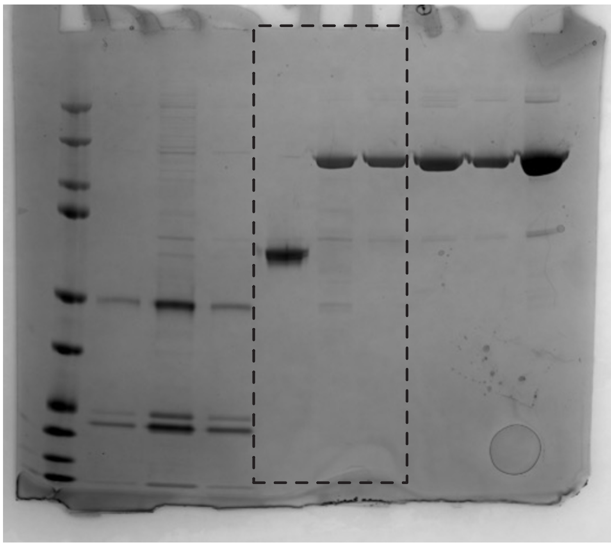

Unmodified EMSA gels of Fig 3c

SYBR gold

IRDye800

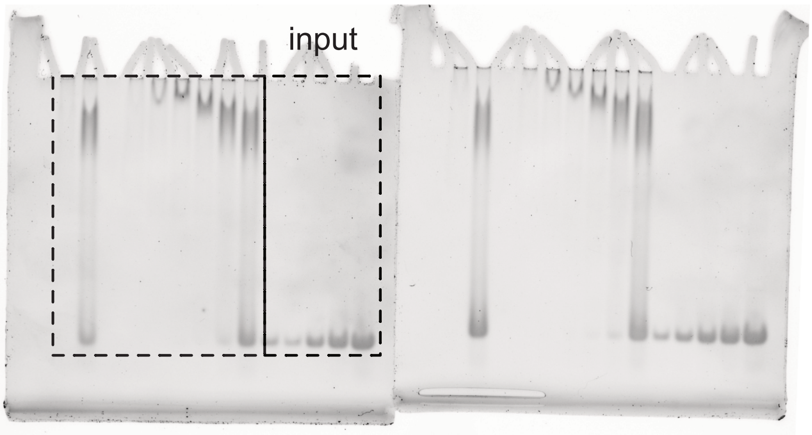

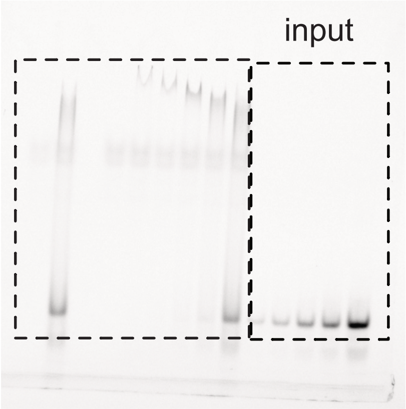

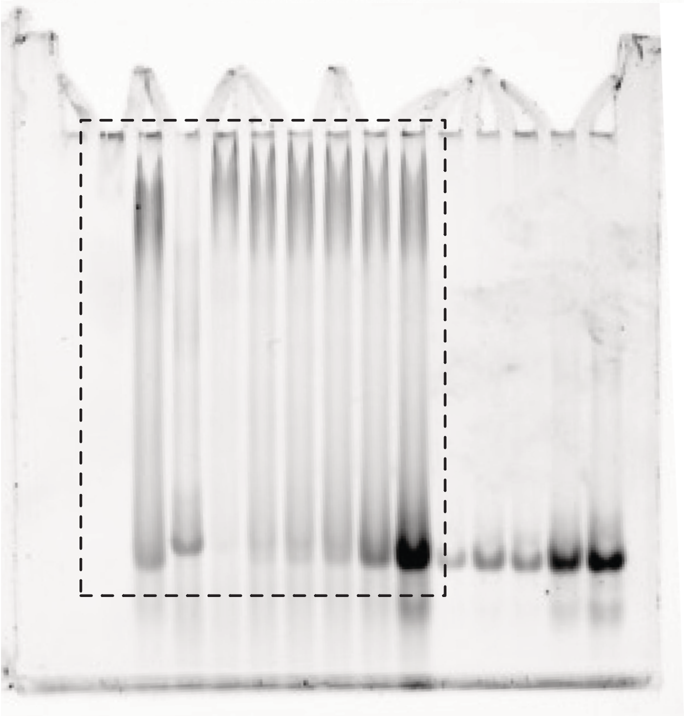

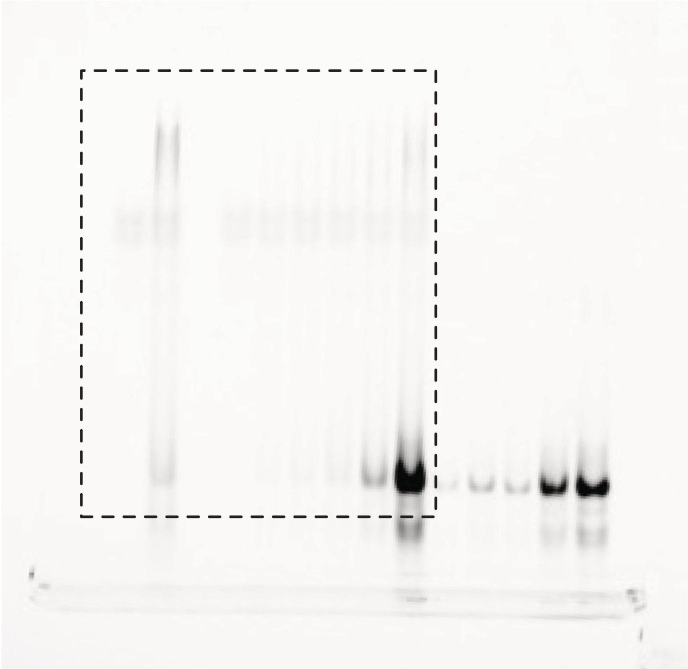

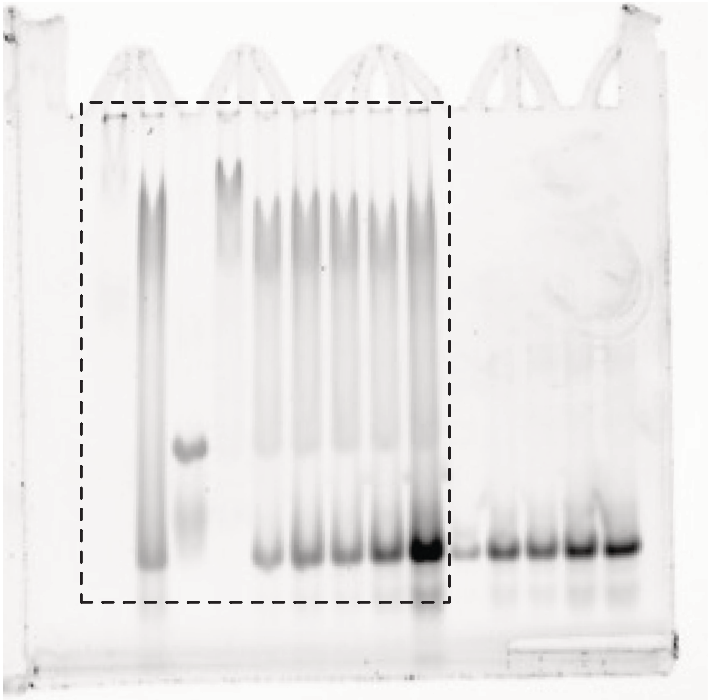

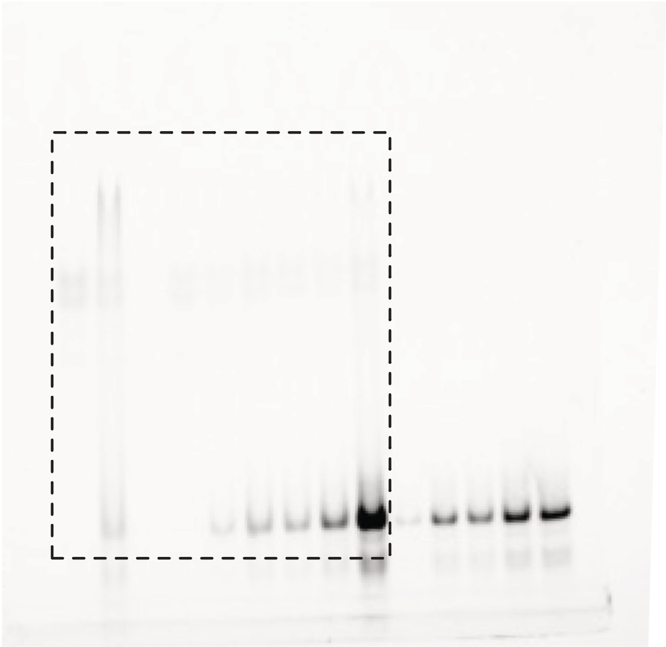

Unmodified EMSA gel of Supp Fig 3a

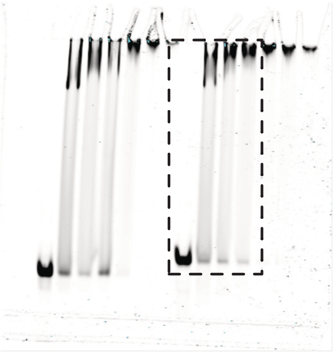

Unmodified EMSA gels of Supp Fig 3b

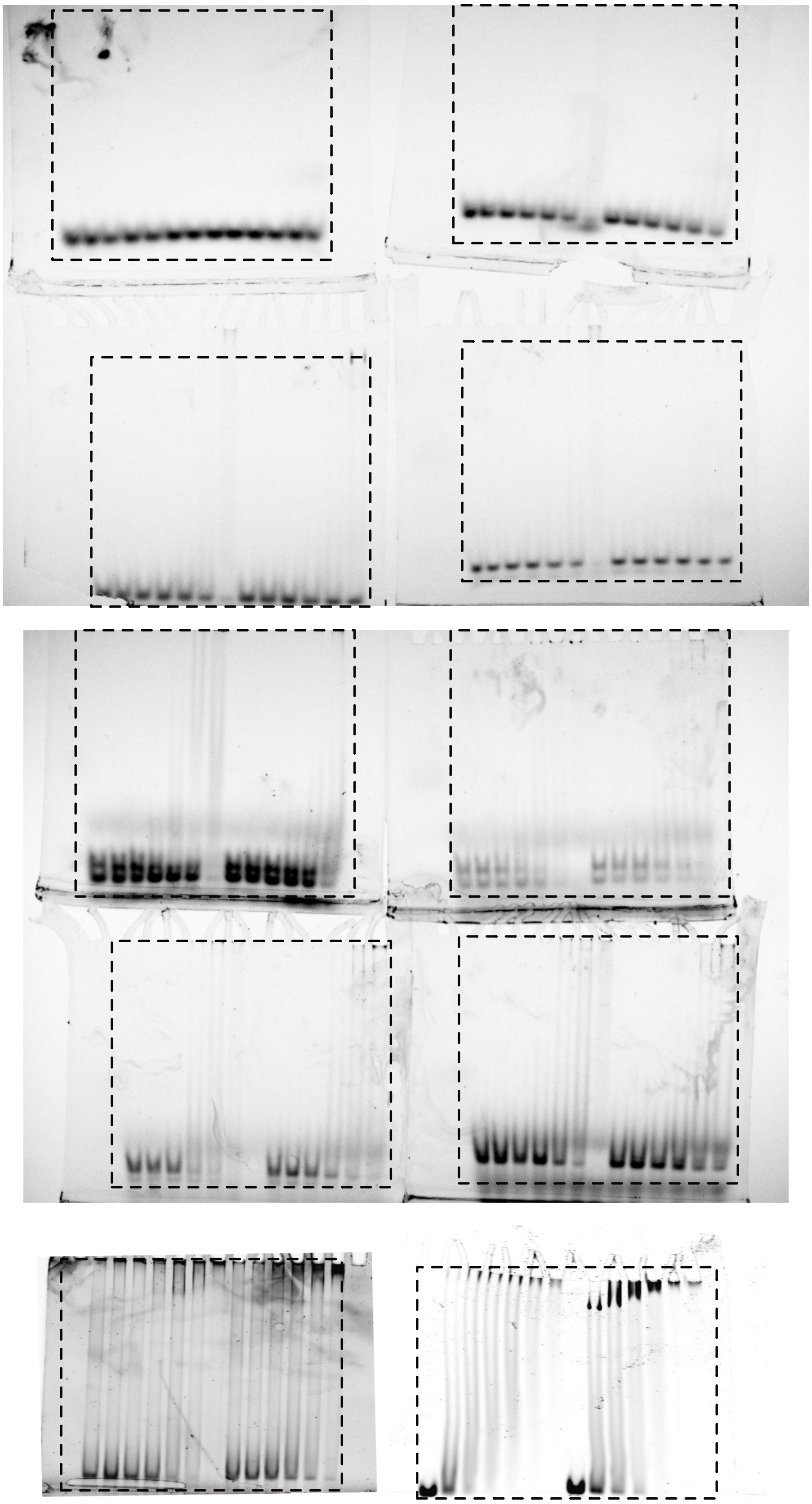

14bp

10bp

24bp

19bp

34bp

29bp

44bp

39bp

90bp

60bp

Unmodified EMSA gel of Supp Fig 4a

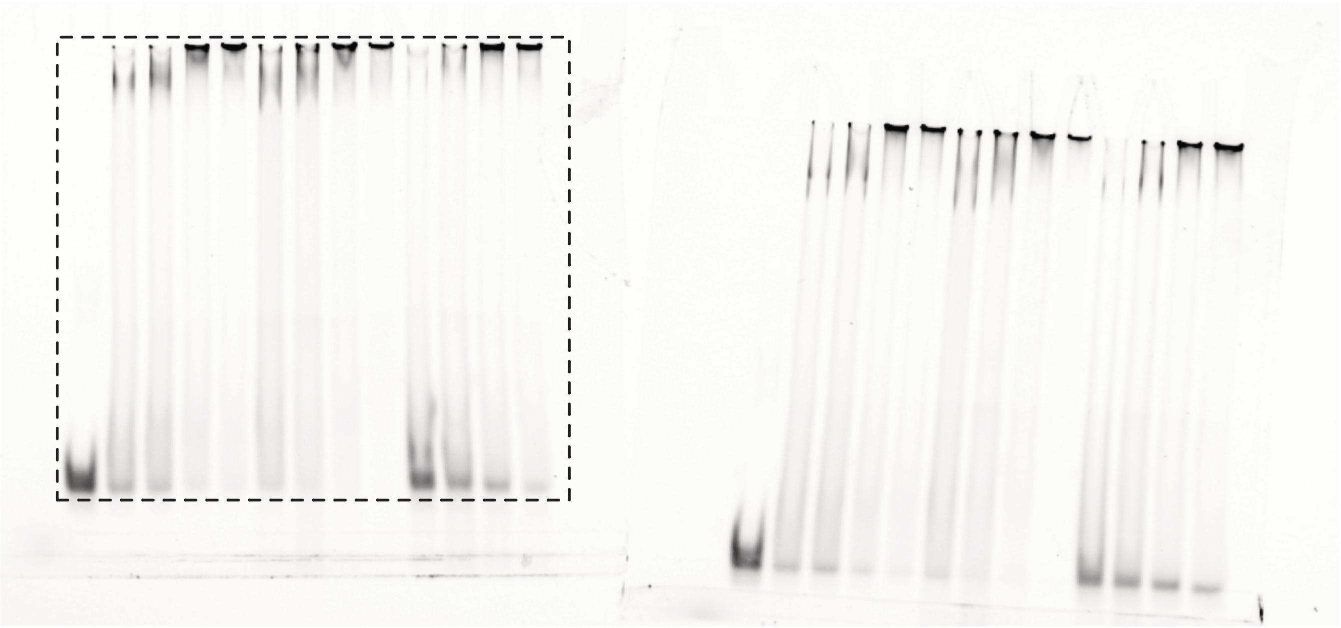

Unmodified EMSA gel of Supp Fig 4d

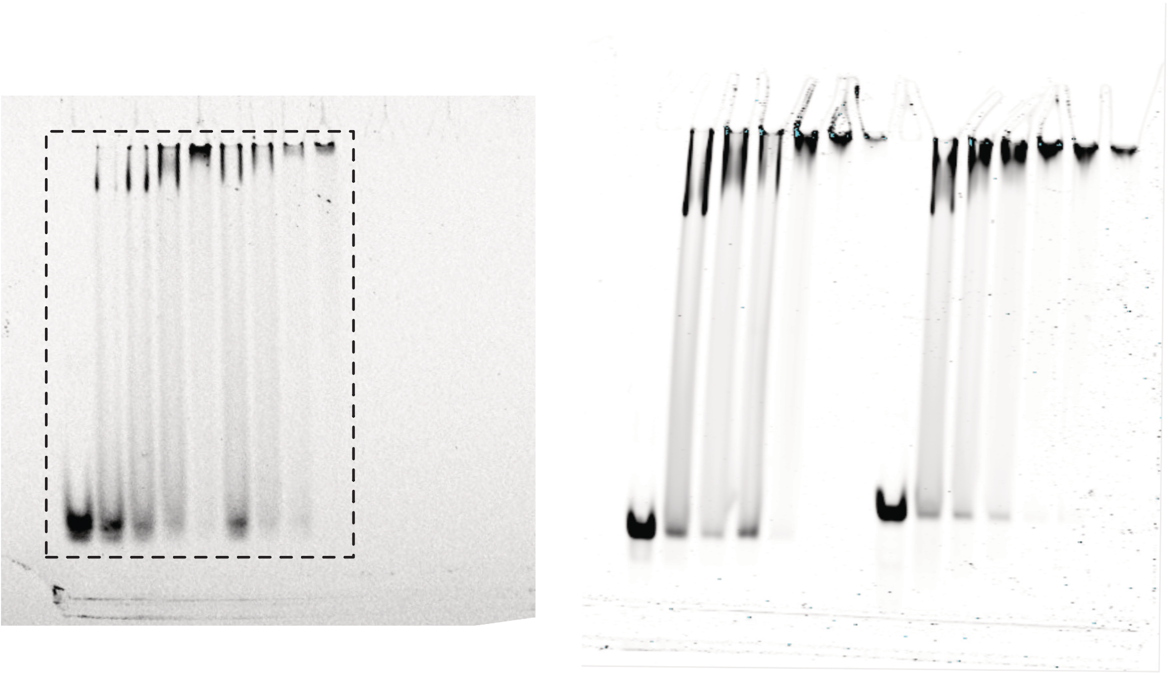

Unmodified EMSA gel of Supp Fig 5e

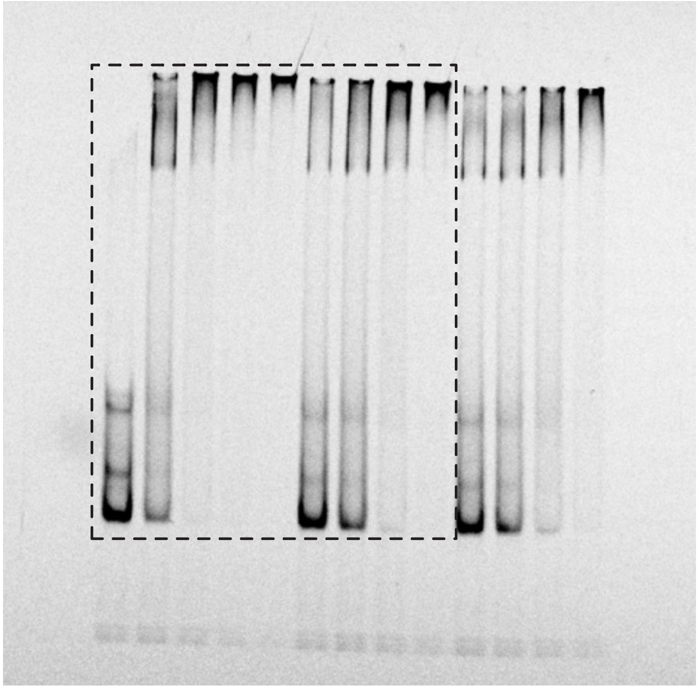

Unmodified EMSA gels of Supp Fig 9a

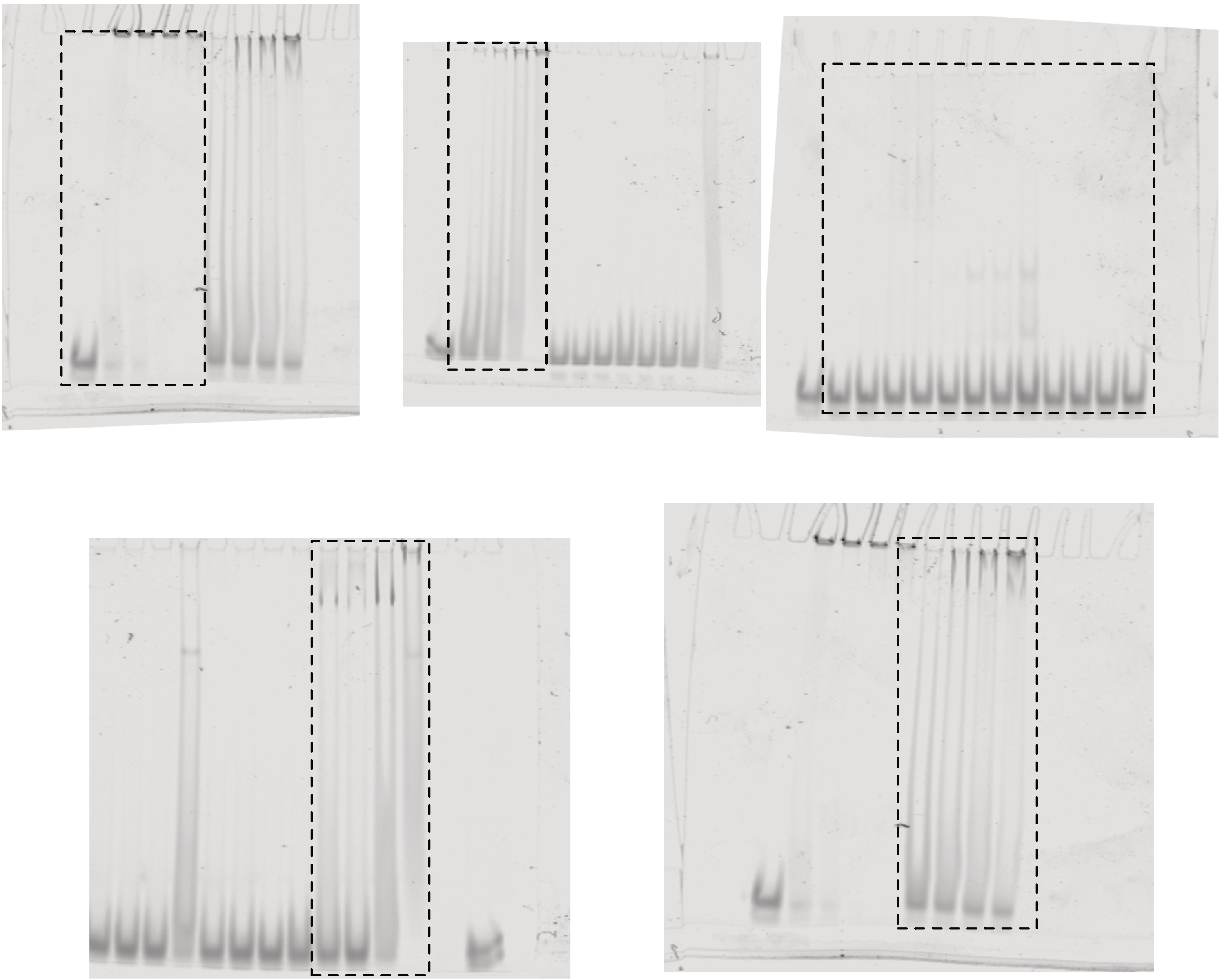

Unmodified EMSA gel of Supp Fig 9b

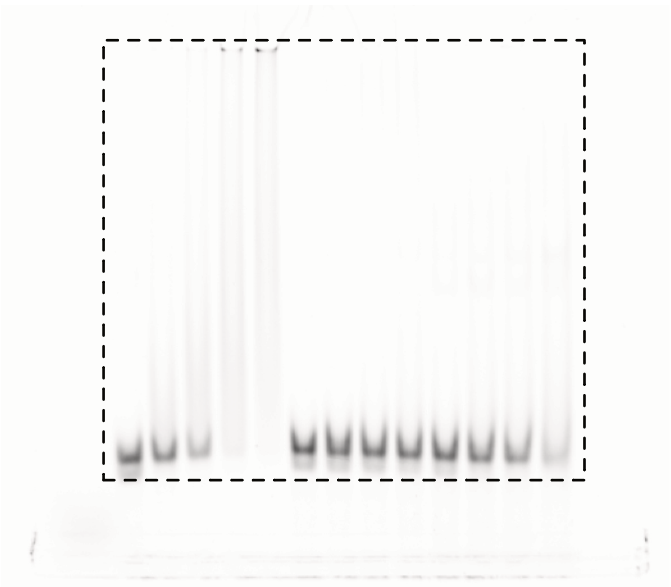

Unmodified EMSA gels of Supp Fig 10a

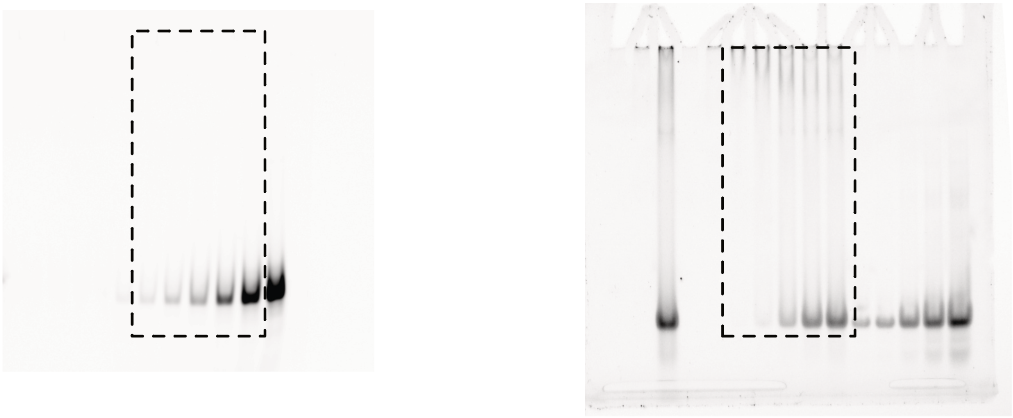

Unmodified EMSA gels of Supp Fig 10b

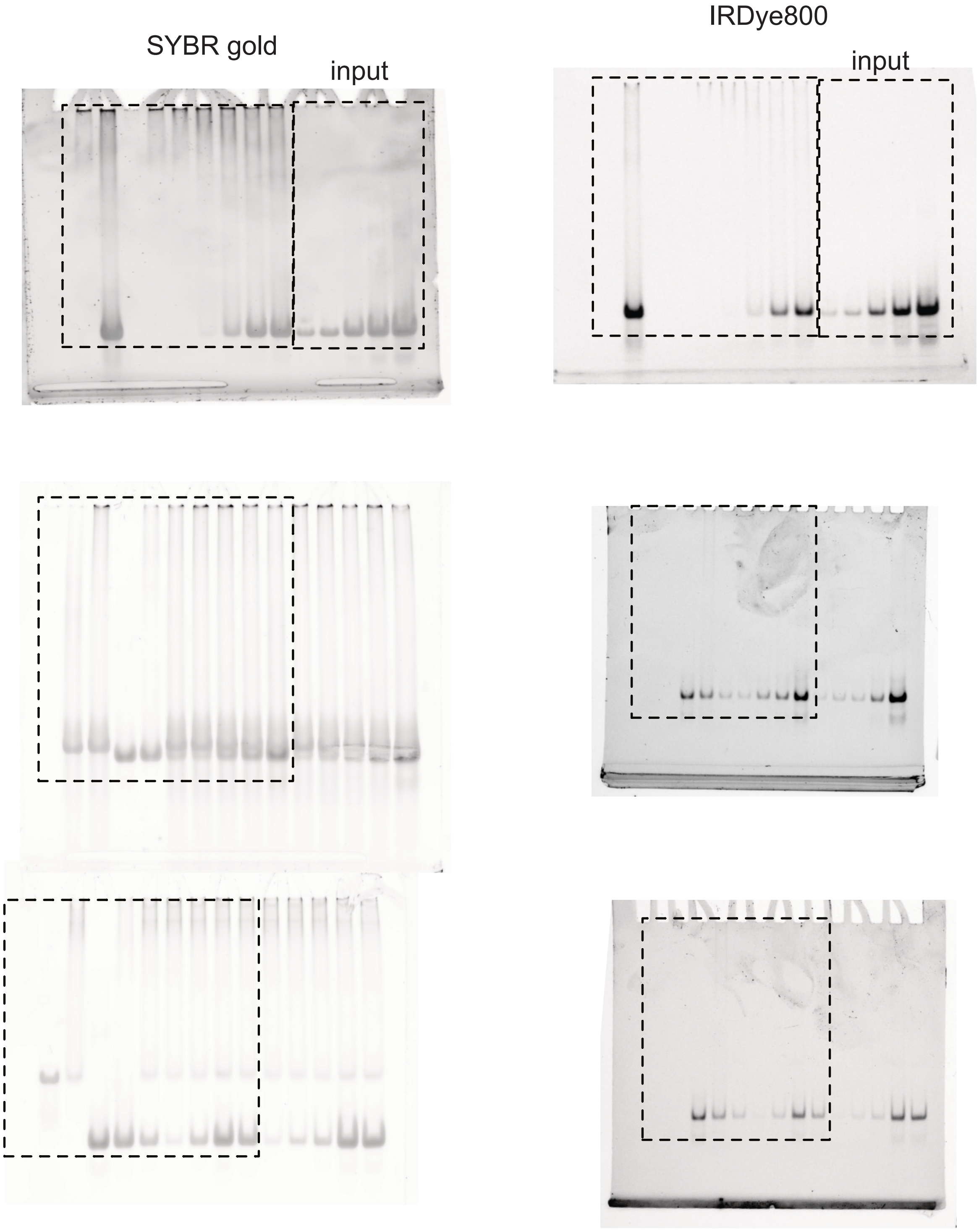

Unmodified western blot of Supp Fig 11c

*Flipped horizontally

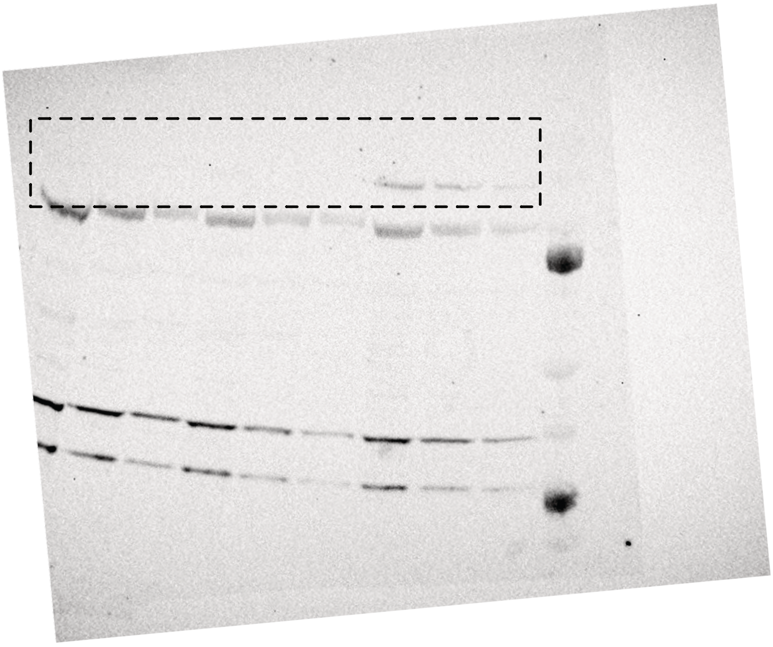

Supplement: Supplementary file 16 — Source Data [file 41467_2025_60751_MOESM16_ESM.zip › Source_data/Source_Data_Fig.pdf]
